# ROS Equilibrium in the Digestive Fluid of the *Nepenthes × ventrata* Trap at Various Developmental Stages

**DOI:** 10.3390/ijms27198462

**Published:** 2026-09-23

**Authors:** Agnieszka Wal, Pawel Staszek, Klaudia Jusińska, Bartłomiej Dominiak, Patrycja Witek, Agnieszka Gniazdowska, Urszula Krasuska

**Affiliations:** 1 Department of Botany and Plant Physiology, Institute of Biology, Warsaw University of Life Sciences, Nowoursynowska 159, 02-776 Warsaw, Poland; pawel_staszek@sggw.edu.pl (P.S.); s215338@sggw.edu.pl (P.W.); agnieszka_gniazdowska@sggw.edu.pl (A.G.); urszula_krasuska@sggw.edu.pl (U.K.); 2 Department of Nanobiotechnology, Institute of Biology, Warsaw University of Life Science, Nowoursynowska 159, 02-776 Warsaw, Poland; klaudia_jusinska@sggw.edu.pl; 3 Department of Physiological Sciences, Institute of Veterinary Medicine, Warsaw University of Life Sciences, Nowoursynowska 159, 02-776 Warsaw, Poland; bartlomiej_dominiak@sggw.edu.pl

**Keywords:** carnivorous plants, antioxidants, phenolic compounds, flavonoids, anthocyanins

## Abstract

Carnivorous plants, such as *Nepenthes* (Nepenthaceae), possess a unique feature: the external digestion of animal-origin food. *Nepenthes × ventrata* pitchers are filled with digestive fluid containing: enzymes, small regulatory compounds and reactive oxygen species (ROS), which also act as signalling molecules. The aim of this study was to investigate the pattern of ROS content and to characterise enzymatic and non-enzymatic ROS modulators in the digestive fluid of *N. × ventrata* at various developmental stages of the pitcher. Significant changes were observed in the levels of hydrogen peroxide (H_2_O_2_) and hydroxyl radical (^•^OH) throughout trap development. The highest concentration of H_2_O_2_ was found in aged pitchers, while the highest level of ^•^OH was recorded in freshly opened immature traps. These findings corresponded with superoxide dismutase and class III peroxidase activities. After maturation of the trap, there was an increase in the activity of diamine and polyamine oxidases. We suggest that the production of ROS in the digestive fluid of pitcher plants at different stages of trap development is controlled by the redox modulatory system. These findings provide a basis for further research into how the regulation of ROS content supports trap development and digestive functions.

## 1. Introduction

The genus *Nepenthes* includes 205 species, native mainly to Southeast Asia but also occurring in Madagascar, Sri Lanka, New Caledonia, and Queensland in Australia [1,2]. *Nepenthes × ventrata* is a natural hybrid between *N. ventricosa* Blanco and *N. alata* Blanco. *N. × ventrata* and its parental species are endemic to forests in the Philippines [2,3]. *Nepenthes* have modified leaves whose structure is compartmentalised. The proximal part is flattened and serves mostly as a photosynthetically active organ. However, the most characteristic feature of *Nepenthes* is a passive pitfall trap formed at the end of the leaf midrib (the distal part of the leaf) [4]. As was demonstrated in *N. khasiana*, the pitcher formation occurs early in leaf development, when the lamina outgrowth is inhibited and the tendril formation is promoted [5]. Depending on the species and environmental factors, the growth of the trap takes several weeks before it opens. Then, the lifespan of the traps ranges from a few weeks to nine months [6]. A typical trap consists of the tendril, which supports the main part (an external “stomach”), a peristome, a collar rim of the trap, and the lid located over the opening. Inside, the trap is divided into the waxy and digestive zones [7,8]. Evolutionarily, the formation of the trap has a background linked to the defence mechanisms and is accompanied by the cooperation of various genes, including those involved in plant flowering [9]. Carnivorous plants use typical stress-induced mechanisms to meet nutritional needs and survive. These mechanisms include not only the emission of various chemical signals to lure prey but also the secretion of pathogenesis-related (PR) proteins and defence-response hormones such as jasmonic acid [10]. Thus, the trap represents a key adaptation of higher plants to an unfavourable environment and is essential for carnivory. *Nepenthes* pitchers are formed from the leaves in which the adaxial surface folds inwards and fuses as the inner wall of the trap is shaped [11]. The first stage of trap development begins with the formation of a small structure at the end of the tendril (the connection of the pitcher to the leaf blade), which progressively enlarges (Figure 1). This is followed by elongation of the pitcher itself, partial unfolding of the peristome, and elevation of the lid (Figure 1) [12]. Trap maturation is accompanied by pitcher colour changes (from mostly green to red), particularly in the waxy zone. Simultaneously, the lid opens, and the peristome fully unfolds (Figure 1) [5]. The rate of pitcher development, as well as the shape formation and colour, varies among species [5,12].

The digestive fluid contains various proteins, including hydrolytic enzymes like proteases, phosphatases, ribonucleases, chitinases, and esterases. These enzymes exhibit high activity and considerable stability over a broad range of pH and temperatures, representing an adaptation to the conditions prevailing inside the pitcher [13,14]. In addition to proteins, in the digestive fluid, various low-molecular components, such as calcium, potassium, magnesium, manganese, sodium, and iron, as well as chloride, bromide, and sulphate anions, have been identified [15]. Their role in prey digestion has not yet been fully elucidated; however, they may contribute to regulating fluid pH and osmotic potential and may also influence enzyme stability and activity. Certain elements, e.g., iron and manganese, may additionally be absorbed by the pitcher tissues and utilised as enzymatic cofactors, thereby supporting both digestive processes and the subsequent assimilation of mineral nutrients [16,17]. The complex and dynamic composition of the digestive fluid indicates that it represents a specialised biochemical environment in which digestive processes are closely linked to redox activity [7]. Moreover, in the pitcher fluid, reactive oxygen species (ROS) and reactive nitrogen species have been detected [3,18,19]. The food digestion performed by pitcher plants is a modification of typical mineral nutrition and, as a physiological process, may be compared to the gastrointestinal processing in animals [7,20]. In higher organisms, ROS are considered as compounds of bimodal function, whose current and local actions depend on the specific molecule chemistry and concentration. They are generated inside the cell, in various compartments, but are also formed outside the cell (in the apoplastic space) [21,22]. Thus, ROS and their modulation system (pro-oxidants and antioxidants) are strictly coupled. Free radicals such as hydroxyl radical (^•^OH), superoxide anion (O_2_^•−^), and non-radical hydrogen peroxide (H_2_O_2_) are directly or indirectly involved in ROS signalling in vascular plants [21]. It is not ROS, per se, but rather, alteration or fluctuation of their concentration, which is responsible for the regulation of different processes. Furthermore, an elevated ROS level is not always harmful to the cell; the reactivity depends on many factors, including the chemical type of ROS molecule, the presence of iron or copper, and the direct biomolecule target [23]. As various ROS react with proteins, lipids, and nucleic acids (not always recognised as a toxic action), their levels are strictly regulated. In the apoplast, polyamine oxidase (PAO) and diamine oxidase (DAO) catalyse amine catabolism with the formation of H_2_O_2_ as a by-product. Peroxidases of class III (POx), depending on the environment and the presence of reductants, remove H_2_O_2_ or catalyse the reaction of ROS formation [24]. The intracellular enzymatic antioxidants are diverse isoforms of superoxide dismutase (SOD) [25]. Compounds with a thiol group (-SH) in the cysteine (Cys) residues are also responsible for ROS scavenging; they belong to the low-molecular-weight thiols and to the proteins/peptides’ thiols [22,26,27]. The presence of such thiols in the trap fluid of *N. × ventrata* during food digestion has been demonstrated [3]. However, there is no information about the possible alteration in the thiol-containing compounds in the digestive fluid during the development of the trap. More data concern changes in the content of phenolic compounds (PCs) in trap tissues and the fluid of carnivorous plants [28,29]. In higher plants, PCs are classified as specialised metabolites involved in many physiological processes [30,31]. They function as antioxidants and pro-oxidants, depending on the pH and the presence of reductants and metal ions [32]. Flavonoids, the predominant PCs in plants, are among the most effective non-enzymatic ROS scavengers [33]. They are also involved in colour development (together with anthocyanins) of different plant organs, thus participating in the plant–animal interaction [29]. PCs, flavonoids, and anthocyanins may serve in ROS formation and scavenging [33].

The colour of the trap changes at various stages of development, and plays distinct roles in prey attraction [6,34]. Pitchers’ trap tissues are green (at least partially), as they contain chlorophyll, but the photosynthetic activity is low, as was demonstrated for *N. alata* and *N. mirabilis* [35]. An interesting exception is *N. pudica*, a species found in North Kalimantan, Indonesia, which produces lower, subterranean, almost white traps [36]. In the trap tissues, flavonoids and anthocyanins have been identified, and their roles are not only to lure the prey but also to protect the plant [29].

Based on the results of our previous work [3,19], we conclude that ROS (especially alterations in ROS levels) are crucial in the trap physiology. The maintenance of ROS homeostasis during trap development requires molecular engagement. Thus, the aim of this work was to investigate components of the ROS modulating system in the digestive fluid during key developmental milestones of the carnivory organ of *N. × ventrata* and during food digestion. We focused on the digestive fluid and glandular zone of the trap, the beneficial part related to plant nutrition. The examination concerned activities of SOD, POx, PAO and DAO. Additionally, we analysed the contents of anthocyanins, carotenoids and chlorophyll *a* and *b* in the glandular (digestive) zone, together with the localisation of flavonoids and PCs, to assess the fluctuation of the content of these compounds, which could potentially be secreted into the digestive fluid.

## 2. Results

### 2.1. Photosynthetic Pigment Concentration in the Pitcher Tissue Depends on the Developmental Stage of the Trap

The lowest chlorophyll *a* content was observed in the glandular zone tissue of closed and freshly opened immature pitchers (Table 1). In mature non-fed and mature fed pitchers, chlorophyll *a* concentration was almost 2-fold higher than in closed and freshly opened immature ones. The highest content was noted in aged pitchers, and it was 0.35 µmol g^−1^ FW (Table 1).

Closed and freshly opened immature pitchers showed the lowest chlorophyll *b* content in the glandular zone tissue, approximately 0.03 µmol g^−1^ FW (Table 1). In mature non-fed and mature fed pitchers, chlorophyll *b* content was about 3-fold higher than in freshly opened immature pitchers. The highest content was detected in aged pitchers (Table 1).

Carotenoid content was at a similar level in closed and freshly opened immature pitchers (Table 1). In mature non-fed pitchers, it was almost 2-fold higher than in closed pitchers, but in mature fed pitchers it remained at a level similar to that observed in closed and freshly opened immature pitchers. The highest carotenoid content was in aged pitchers (approximately 0.39 µmol g^−1^ FW) (Table 1).

In closed and freshly opened immature pitchers, anthocyanin content was at a similar level of approximately 0.160 µmol g^−1^ FW (Table 1). In mature non-fed pitchers, it was almost 2-fold higher. In mature fed and aged pitchers, anthocyanin content was the same as that observed in closed and freshly opened immature pitchers (Table 1).

### 2.2. Hydroxyl Radical (^•^OH) Level in the Digestive Fluid Increases Transiently in the Opened Immature Trap

The highest ^•^OH level was detected in the digestive fluid of freshly opened immature pitchers, where it reached approximately 5.1 nmol NO_2_^−^ trap^−1^ (Figure 2A). In the fluid collected from closed pitchers, the ^•^OH level was approximately 35% lower than in freshly opened ones. In mature non-fed and mature fed pitchers, the ^•^OH level was markedly lower. The lowest ^•^OH level was observed in the digestive fluid from aged pitchers showing signs of senescence, where its concentration was 5-fold lower than in freshly opened immature traps (Figure 2A).

### 2.3. H_2_O_2_ Concentration in the Digestive Fluid Increases During the Development of the Trap, Reaching the Highest Value in Pitchers During Senescence

The lowest H_2_O_2_ content was in the digestive fluid of closed pitchers (around 1.5 pmol H_2_O_2_ trap^−1^) (Figure 2B). In the fluid collected from freshly opened immature pitchers, H_2_O_2_ content was approximately four times higher than in the closed ones. In mature non-fed and mature fed pitchers, H_2_O_2_ content was at the same level of about 15 pmol H_2_O_2_ trap^−1^. The highest H_2_O_2_ content was in the digestive fluid of aged pitchers, around doubled as compared to mature fed ones (Figure 2B).

### 2.4. SOD Activity in the Digestive Fluid Exhibits a Wave-like Pattern During Pitcher Development

The lowest SOD activity was determined in the digestive fluid of closed and mature non-fed pitchers, where the values were approximately 0.50 U min^−1^ trap^−1^ (Figure 3A). In the fluid collected from freshly opened immature pitchers, SOD activity increased transiently and was approximately 4-fold higher than in closed and mature non-fed pitchers. The highest SOD activity was in the digestive fluid of mature fed pitchers, where it reached approximately 2.2 U min^−1^ trap^−1^, while in aged pitchers, its activity decreased slightly and was around 15% lower than in mature fed pitchers (Figure 3A).

### 2.5. POx Activity in the Digestive Fluid Fluctuates During the Trap Development

The highest POx activity was determined in the digestive fluid of closed pitchers, where it was approximately 1.6 nmol purpurogallin min^−1^ trap^−1^ (Figure 3B). The lowest POx activity was measured in mature non-fed and in aged pitchers. In the fluid collected from freshly opened immature pitchers, POx activity was approximately 44% lower than in closed ones. In the mature fed pitchers, POx activity in the fluid was more than 2-fold higher than in the fluid from mature non-fed or aged traps (Figure 3B).

### 2.6. PAO and DAO Activities Increase in the Digestive Fluid During the Development of the Trap, Exhibiting the Transient Decline in the Fluid of the Mature Fed Pitcher

The lowest PAO activity was measured in the digestive fluid in closed pitchers (Figure 3C). In opened immature pitchers, PAO activity in the fluid was approximately 3-fold higher than in closed pitchers. In the fluid from mature fed pitchers, PAO activity was only slightly (16%) but significantly lower than in mature non-fed pitchers (Figure 3C). The highest PAO activity was in the fluid from aged pitchers, showing signs of senescence (Figure 3C).

The pattern of DAO activity in the digestive fluid during the trap development was similar to that observed for PAO activity, although its value was even higher for mature non-fed and aged pitchers (Figure 3C). The lowest DAO activity was found in the fluid from closed pitchers. In the fluid from opened immature pitchers, DAO activity was approximately 4-fold higher than in closed pitchers. It increased in mature non-fed pitchers and declined almost twice in the fluid of the mature fed pitchers (Figure 3C). The highest DAO activity was observed in the fluid from aged pitchers (Figure 3C).

### 2.7. Phenolic Compounds (PCs) Are Present in the Glandular Zone of the Trap at All Stages of Its Development

PCs were detected in the glandular zone of pitchers at all analysed developmental stages (Figure 4). The staining was mainly localised in the digestive glands, indicating the accumulation of PCs in these structures. In closed immature and freshly opened immature pitchers, the colouration corresponding to PCs was visible but less intense. A stronger staining signal was observed in mature opened non-fed and mature opened fed pitchers, as well as in aged pitchers (Figure 4).

### 2.8. Flavonoid Concentration in the Digestive Fluid Increases in Matured Traps

The lowest flavonoid content was detected in the digestive fluid of closed and freshly opened immature pitchers, where it reached 4.49 and 8.12 µg quercetin trap^−1^, respectively (Figure 5A). In the digestive fluid of mature non-fed, mature fed, and aged pitchers, a similar flavonoid content level was observed, and it was significantly higher (3–6-fold) than in the fluid from closed and freshly opened immature pitchers (Figure 5A).

### 2.9. Flavonoids Are Localised in the Glandular Zone of the Trap

Confocal imaging after DPBA staining revealed the presence of flavonoid-associated fluorescence in the glandular zone of pitcher traps at all analysed developmental stages (Figure 5B,C). In unstained control tissues, only weak autofluorescence was observed (Figure 5B). Stained DPBA tissue sections and epidermal peels exhibited markedly stronger fluorescence within glandular structures (Figure 5C). In closed and opened immature traps, the signal was present but relatively weak. The strongest fluorescence was observed in fully developed fed traps, where the signal occupied a large part of the glandular tissue. In aged traps, the fluorescence signal remained detectable, but was unevenly distributed throughout the gland.

### 2.10. Reducing Capacity of the Digestive Fluid Fluctuates During Trap Development with Lower Values in Mature Fed Traps

The highest reducing capacity was detected in the digestive fluid of freshly opened immature pitchers, where it reached approximately 35% (Figure 6A). In the fluid collected from closed and mature non-fed pitchers, the reducing capacity was only slightly lower. Feeding of the trap resulted in a further decline of reducing capacity in the digestive fluid, which was the lowest (around 18%) in mature fed pitchers. In aged pitchers, the reducing capacity in the digestive fluid increased slightly and reached around 23% (Figure 6A).

### 2.11. The Concentration of the Compounds with -SH Groups in the Digestive Fluid Increases During Trap Development

The content of compounds with -SH groups determined without TCA precipitation was the lowest in the digestive fluid of closed and freshly opened immature pitchers, where it was approximately 4 µmol TNB trap^−1^ (Figure 6B). In the fluid from mature non-fed, mature fed, and aged pitchers, the content of compounds with -SH groups was approximately 2-fold higher than in closed and freshly opened immature pitchers (Figure 6B).

After TCA protein precipitation, the content of compounds with -SH groups was markedly lower in all analysed samples (Figure 6B). The highest content of low-molecular-weight thiol compounds was detected in the digestive fluid of mature fed pitchers, where it reached approximately 4.6 µmol TNB trap^−1^. In the fluid from pitchers at other developmental stages, the values of this parameter were much lower and ranged from around 0.5 to 1.2 µmol TNB trap^−1^ (Figure 6B).

## 3. Discussion

Carnivorous plants are mixotrophs, which developed an adaptation based on plant–animal communication, as well as defence mechanisms against biotic stressors [10,37]. A key attribute of carnivory is the ability to form a trap, and in *Nepenthes*, this organ has evolved from a flat, typical leaf into a structurally complex, multi-functional trap—a pitcher [4]. The environment created inside the trap of carnivorous plants is unique, resembling both the extracellular space [3] and the rhizosphere [38]. The traps serve to provide mineral nutrition to the plant, a function typically performed by the roots [39]. This specific environment may be regarded as the apoplastic zone, which contains diverse components like proteins, sugars, ions, amino acids, PCs, and organic acids [40]. The interior of the pitcher trap is also known as phytotelmata, a water tank enclosed by living terrestrial plants’ tissues inhabited by the so-called inquilines [41]. The trap’s complex structure and function are examples of the plasticity of gene expression [9] and the action of growth regulators [42], which enable oriented cell division and alterations in growth rates. Our experimental model encompasses several developmental stages of the trap: pre-opening, immediately post-opening (maturing trap), mature trap, and a trap showing signs of ageing on the lid. Additionally, for the mature trap, we considered two physiological states: before the onset of digestion (non-fed) and during digestion (fed).

In higher plants, ROS (besides hormones) are engaged in the regulation of growth and development [43]. ROS homeostasis requires numerous molecular systems to maintain the current level necessary for proper metabolism. ROS presence and participation in food digestion in carnivorous plants, including pitcher plants, has been demonstrated [3,18,19,44], whereas the role of ROS in the development of *Nepenthes* traps remains unexplored. Previously, we have shown alterations in O_2_^•−^ generation in the digestive fluid of *N. × ventrata* at various developmental stages of the trap, and during the digestion of food [19]. In this study, we examined the highly reactive and extremely unstable hydroxyl radical (^•^OH). This oxidant is mostly known for its ability to damage the majority of organic compounds. On the other hand, it takes part in the regulation of many physiological processes, including defence mechanisms and cell death [21,45]. The highest levels of ^•^OH in the *N. × ventrata* trap fluid in the closed and just-opened pitchers have been detected (Figure 7). These results agree with the level of O_2_^•−^ in the fluid of the closed trap [19]. In the fluid, some of the radicals are probably formed from H_2_O_2_, in the presence of POx. The lowest H_2_O_2_ content was shown in the fluid from traps at early developmental stages, characterised by the highest POx activity. The extracellular release of ROS was noticed, for example, during seed germination [46], where radicals facilitate cell wall loosening and seed embryo radicle protrusion [47]. At the early stage of pitcher trap development, ^•^OH and O_2_^•−^ may participate in the promotion of growth of this organ. Additionally, ^•^OH is involved in stomatal opening (at low concentration) or closure (at high concentration) in *Arabidopsis thaliana* (L.) Heynh. [48]. Stomata movements depend on solute accumulation, and turgor pressure; thus, ^•^OH (as we noted its low levels) in the digestive fluid of *N. × ventrata* may participate in the regulation of the fluid level inside the trap. However, this hypothesis must be experimentally verified. Also, free radicals may prevent the uncontrolled growth of microorganisms, as we assumed before [19].

Despite the presence of free radicals in the trap fluid, oxidants are under the control of various reductants. In the first three phases of trap development, the digestive fluid of *N. × ventrata* is characterised by a relatively high reducing capacity. Given that in closed and in freshly opened immature traps’ fluid, low-molecular compounds containing -SH groups are less abundant, and levels of flavonoids (Figure 7) and PCs [19] are also low, other reductants must play a protective role. The presence of reduced glutathione (GSH) in the “green stomach” of the Venus flytrap (*Dionaea muscipula* (J.Ellis)) was noted after stimulation with coronatine, a structural and functional analogue of jasmonate [49]. However, the authors were not able to determine the ascorbate, a common and abundant plant low-molecular antioxidant. Ascorbate is an essential antioxidant in the apoplastic space [40]; thus, its presence in the digestive fluid should not be ruled out.

On the other hand, in higher organisms, free radicals (especially at physiological levels) are now considered more as “friends than foes” [22]. Since they are required, they must be constantly produced, even without a stimulus. At the early developmental stage of the trap, digestion or other processes that could stimulate ROS formation in the fluid do not occur. In this case, ROS production may be regulated by the molecules secreted from the trap tissue. Among them, good candidates are plant secretory POxs (EC 1.11.1.7; class III peroxidases), heme-containing enzymes that belong to the peroxidase-catalase superfamily [50,51]. Depending on the presence of specific substrates, POxs may act as antioxidants or pro-oxidants [45]. One-electron oxidation of, e.g., PCs by these enzymes leads to H_2_O_2_ reduction to water [52]. On the contrary, when POx uses thiols or salicylic acid as a substrate, the derivative radicals may react with O_2_, and then free radicals are formed [51]. The presence of POx in *N. × ventrata* digestive fluid has been verified [53]. In this study, we have demonstrated high POx activity in the fluid of closed *N. × ventrata* traps. At the same developmental stage of the pitcher, low contents of total PCs [19] and total flavonoids (Figure 7) were noted. We also confirmed the presence of the compounds with -SH groups (especially low-molecular thiols) in the fluid of the closed trap (Figure 7). Thus, we conclude that POx may function as a pro-oxidative enzyme, especially when the trap is intensively growing, before opening and maturation. Moreover, POxs may be constitutively expressed in the trap’s tissues, and secreted into the digestive fluid, as they are multi-purpose proteins involved in many physiological processes. In *N. khasiana*, transcripts encoding POx were detected in both closed and open pitchers [54], which points to constant POx synthesis. We have previously demonstrated that during food digestion by *N. × ventrata*, POx activity in the trap fluid was stimulated [3]. In this study, we confirmed that in the mature, fed pitchers, POx activity was more than 2-fold higher than in the mature pitchers before food introduction. Thus, we conclude that POx also participates in food digestion (maybe as a pro-oxidant, depending on the substrate availability). This observation is consistent with the findings of Hatano and Hamada [55], who demonstrated that the addition of chitin to the digestive fluid of *N. alata* induced the secretion of POx. The feeding-induced increase in POx activity may therefore represent a pitcher response to the prey and contribute to the regulation of ROS generation during digestion. Moreover, as we introduce the proteins from the egg white solution (without chitin), an increase in POx activity may be caused by the specific products of digestion (e.g., various nitrogen-containing compounds) [56,57].

Following the application of food into *N. × ventrata* pitchers, decreased O_2_^•−^ production and an increase in the free radical scavenging capacity of the digestive fluid were previously observed [19]. The rapid dismutation of O_2_^•−^ to H_2_O_2_ and O_2_ is catalysed by SOD [43]. Among various isoforms of SOD, Cu/ZnSOD is the most active in the apoplast, especially as part of the defence mechanism against different stressors [58]. In the fluid of mature fed pitchers of *N. × ventrata*, SOD activity was approximately 5-fold higher than in the mature non-fed pitchers (Figure 3A). The feeding-induced increase in SOD activity lowered O_2_^•−^ level by accelerating its dismutation to H_2_O_2_ (Figure 2B). At this time point, H_2_O_2_ could subsequently be used by POx, whose activity was also enhanced (Figure 3B). As we suggested before, digestion occurs in the presence of microorganisms; thus, radical content may be restricted to protect symbionts [3]. In the digestive fluid of mature non-fed traps, the contents of total PCs [19] and total flavonoids (Figure 7) were high. Despite the activation of POx and SOD, the H_2_O_2_ and ^•^OH levels in the digestive fluid did not differ between mature fed and mature non-fed pitchers of *N. × ventrata*. This points to the signalling role of ROS, especially H_2_O_2_ [22], as we previously proposed [3]. ROS are engaged in alterations of the membrane potential (electrical signal/ROS wave) during plants’ responses to numerous stimuli [59,60,61]. Carnivorous plants (e.g., *Drosera*) use electrical signals for nutritional purposes [49,62]. However, data concerning the ROS wave in the traps of pitcher plants are missing. In our study, the highest H_2_O_2_ content in the digestive fluid of *N. × ventrata* was detected in the pitchers exhibiting signs of senescence and was accompanied by relatively high SOD and low POx activity (Figure 7). A similar relationship between H_2_O_2_ accumulation, changes in antioxidant enzyme activities, and the progression of senescence has been observed in the leaf tissues of other plant species. During maize (*Zea mays* L.) leaf senescence, SOD activity decreased by approximately 1.5-fold, whereas H_2_O_2_ content was approximately 6-fold higher than in young leaves [63]. Treatment of tomato (*Solanum lycopersicum* L. cv. “Micro-Tom”) leaves with H_2_O_2_ accelerated senescence, as evidenced by enhanced chlorophyll degradation [64]. Exposure to H_2_O_2_ also disturbed redox homeostasis and increased ROS production. The formation of O_2_^•−^ was almost 1.5-fold higher, whereas POx activity decreased by nearly 30% [64]. Ageing of the trap of *N. × ventrata* was not accompanied by a decrease in the chlorophyll *a*, chlorophyll *b*, and carotenoid content in the glandular zone. On the contrary, the highest concentrations of chlorophyll *a* and chlorophyll *b* were noted, and the ratio of chlorophyll *a* and chlorophyll *b* was almost 1:1. However, the senescence of the trap was not advanced at this stage; it primarily affected the lid and the peristome. Therefore, chlorophyll degradation in the glandular part may not occur at this stage.

The glandular zone is responsible for secretion and absorption of various compounds, including ROS, as we demonstrated before [3]. Thus, this part of the trap is strictly responsible for nutrition. Aged traps slowly lose their ability to digest and absorb food; thus, the physiology of the trap also changes from a metabolically active to a senescent organ. We suppose that during trap ageing, carnivory is no longer the predominant function. Under light conditions, chloroplasts strongly impact mitochondrial metabolism, as this is linked to the oxygenase activity of RuBisCO and nitrogen assimilation [65]. It is possible that the limitation of chlorophyll formation to prevent photosynthesis is no longer essential in tissues of aged *N. × ventrata* traps. Moreover, higher contents of carotenoids in *N. × ventrata* traps, which exhibited visible senescence signs, may be linked not only to photosynthesis but rather to the formation of other metabolites, e.g., ABA or strigolactones [66]. Generally, the trap of *Nepenthes* is the carnivory attribute, which is linked to the low net photosynthetic assimilation rate and low chlorophyll content, as was demonstrated for *N. alata* and *N. mirabilis* [35]. The increase in chlorophyll *a* and chlorophyll *b* concentration was observed in fed *N. talangensis*, compared to the non-fed traps [67]. The authors proposed that the nitrogen absorbed from the food stimulates chlorophyll biosynthesis. In our study, food introduction to the trap resulted in a decrease in chlorophyll *a* and chlorophyll *b* content together with the carotenoid and anthocyanin concentrations in the glandular (digestive) zone. We believe that high mitochondrial respiration and ATP production, even under light conditions, are necessary to maintain the function (proton pumps) of this specific part of the trap, especially during digestion. Photosynthetic activity may interfere with mitochondrial metabolism; thus, it may be limited by restricted chlorophyll synthesis. Moreover, differences in chlorophyll content may exist among various *Nepenthes* species.

The trap is used to lure the prey. We observed an increase in the anthocyanin content in the glandular zone of *N. × ventrata*. Belonging to the flavonoids, anthocyanins, depending on the pH, light and temperature, display different colours, and play a key role in plant–animal interactions, and in ROS scavenging [29].

Another group of metabolites of low molecular weight is flavonoids, which are ubiquitous throughout the plant kingdom [68]. Besides pigmentation, which in the case of carnivorous plants may have an important role in luring prey, they participate in facilitating plant–microbe associations within the rhizosphere or attracting pollinators [68]. Thus, the presence of flavonoids in the carnivorous traps is not surprising [29]. In our study, we demonstrated the presence of flavonoids in *N. × ventrata* digestive fluid at various developmental stages of the trap. The lowest content of total flavonoids was observed in the fluid of closed traps (Figure 7). As mentioned, during this developmental phase, there is no stimulus from the food. The absence of significant differences in flavonoid content in the digestive fluid of mature non-fed and mature fed pitchers suggests that food (protein) decomposition did not affect the total flavonoid content. However, the flavonoid content in the fluid of mature non-fed pitchers was approximately 5-fold higher than in the fluid of freshly opened immature pitchers, indicating their role in pitcher maturation. A similar pattern was previously observed for the total PC content in the digestive fluid of *N. × ventrata* [19]. Histochemical localisation of PCs (Figure 4) demonstrated their presence mainly in the digestive glands. The developed colour was visible in all analysed tissue samples collected from the traps at various stages. However, a more intense signal was observed in the mature fed and non-fed pitchers and in aged pitchers with visible symptoms of ageing. Flavonoid localisation (Figure 5B,C) also revealed intense fluorescence predominantly in the digestive glands, particularly in the mature and senescing tissues. The localisation of PCs and flavonoids, together with their higher content in the fluid of mature pitchers, indicates that the digestive glands may represent an important site of the synthesis and accumulation of specialised metabolites secreted into the fluid. These results further suggest that PC metabolites, including flavonoids, gradually accumulate in the digestive fluid during pitcher maturation. Using high-resolution mass spectrometry, Dávila-Lara et al. [69] showed the presence of flavonoids in pitcher tissues and in the digestive zone. They identified three cyanidin glycosides in the mature pitchers of seven *Nepenthes* taxa. The authors detected transcripts of genes associated with anthocyanin biosynthesis in the pitcher tissues [69]. These findings confirm that flavonoid metabolism is physiologically beneficial to the digestive zone of *Nepenthes* pitchers. Transcriptomic analyses using pitcher tissues at different developmental stages demonstrated the expression of genes encoding key enzymes of flavonoid and anthocyanin biosynthesis, including chalcone synthase, chalcone isomerase, and anthocyanidin synthase [9]. Transcript levels of genes associated with the phenylpropanoid and anthocyanin biosynthetic pathways were generally higher in young pitcher tissues than in mature pitchers. This suggests that the synthesis of these metabolites begins before the pitcher reaches full maturity [9]. Compounds produced during pitcher development may subsequently accumulate gradually in the tissues and be transported into the digestive fluid, resulting in their higher contents in the fluid of mature pitchers.

Thiol-containing compounds of low molecular mass and peptides/proteins are responsible for ROS homeostasis and signalling [22]. The presence of and increase in thiol content in *N. × ventrata* trap fluid during digestion have been demonstrated [3]. In carnivorous plants, such compounds may be involved in redox balance maintenance (including the protection of hydrolytic enzymes) in the digestive fluid [49]. Following protein precipitation with TCA, the content of compounds with –SH groups in the fluid of *N. × ventrata* fed traps was almost 3-fold higher than in the fluid collected at the other developmental stages of the trap (Figure 7). This indicates an increase in low-molecular-weight thiols during digestion, although their origin cannot be definitively linked to the plant. Ovalbumin, the major protein in chicken egg white (which was used as a food in this study), contains four free Cys residues, which are largely buried within the native protein structure, as well as one disulphide bond [70]. Partial unfolding and proteolysis of ovalbumin in the digestive fluid may have increased the accessibility of –SH groups and led to the formation of low-molecular-weight, Cys-containing peptides that were not precipitated by TCA. This can also explain the increase in the concentration of thiol-containing compounds in the digestive fluid at these stages of trap development. Enrichment of compounds with -SH (with and without TCA) in the digestive fluid of aged traps may be due to the presence of undigested proteins or larger peptides (Figure 7). Nevertheless, the presence of low-molecular-weight compounds with -SH groups in closed and freshly opened immature traps suggests the possible secretion of GSH into the digestive fluid to protect proteins or serve as a redox buffering compound.

There is no data on the presence of polyamines in carnivorous plants, but their occurrence is most probable. Polyamines are low-molecular-weight aliphatic compounds with two or more amino groups, synthesised in eukaryotic and prokaryotic cells, and acting in various physiological processes, such as responses to stressors, fruit development, maturation, and senescence [71]. They are exported to the apoplast and affect plasma membrane H^+^ pumps and Ca^2+^ pumps [72]. Products of polyamine catabolism are also the result of plant reaction to various stimuli [72]. Polyamine catabolism is performed by PAO and DAO (or copper amine oxidase—CuAO). The occurrence of multiple PAO and DAO genes and their unique alteration in the genome of aquatic bladderwort (*Utricularia gibba* L.) have been demonstrated [73]. Outside the cell, both oxidases produce H_2_O_2_ as a by-product [74]. In our study, the highest activities of PAO and DAO were detected in the fluid of *N. × ventrata* aged traps (Figure 3C). The fluid collected at this developmental stage of the trap was rich in H_2_O_2_. As polyamine catabolism catalysed by PAO and DAO generates H_2_O_2_, the increased activities of these enzymes may have provided an additional source of H_2_O_2_ in the fluid of aged pitchers (Figure 7). A similar relationship between polyamine catabolism, H_2_O_2_ production, and senescence has been demonstrated in barley (*Hordeum vulgare* L.) leaves [75]. During dark-induced barley leaf senescence, DAO activity increased approximately 2-fold, whereas PAO activity increased approximately 5-fold. This was accompanied by an increase in H_2_O_2_ content [75]. The lowest PAO and DAO activities were detected in the digestive fluid of closed *N. × ventrata*. The increase in PAO and DAO activities during trap development indicates an enhanced capacity of the digestive fluid to oxidise polyamines/bioactive amines, but does not directly demonstrate their endogenous presence in the fluid. If such substrates are present, they may originate from pitcher tissues or from microorganisms living in the trap. Nevertheless, our results indicate that amine oxidation may contribute to ROS modulation, particularly in aged pitchers. Future studies should include direct identification and quantification of polyamines in the digestive fluid.

## 4. Materials and Methods

### 4.1. Experimental Model and Plant Culture

Plants of *Nepenthes × ventrata* Hort. ex Fleming (*N. ventricosa* Blanco *× N. alata* Blanco) were used in the experiment. The study was conducted at Warsaw University of Life Sciences, Poland, where the plants were maintained in a greenhouse under controlled conditions, with relative humidity of 60% and temperature of 28 °C. In spring and summer, plants were grown under natural light conditions, while in autumn and winter sodium lamps were used to provide a 16/8 h day/night photoperiod. The plants were cultivated in pots filled with a mixture of acidic peat, perlite, and sphagnum moss and watered every second day with distilled water.

The experimental model included five stages of trap development (Figure 8): closed immature pitchers, freshly opened immature pitchers (16 h after opening), mature opened non-fed pitchers, mature opened pitchers 1 day after feeding, and pitchers with visible symptoms of senescence (aged). Feeding consisted of adding egg white solution directly to the pitcher fluid. The solution contained 1 µg protein µL^−1^ and was applied in a volume of 40 µL pitcher^−1^. The digestive fluid and trap tissue were collected from each developmental stage. The volume of the digestive fluid collected from each pitcher was measured individually. For analyses performed using the digestive fluid, the amount of the analysed compound was calculated for the total volume of the fluid collected from the corresponding pitcher and expressed per trap. One pitcher collected from one individual plant was considered one biological replicate. For each developmental stage, 4–6 biological replicates were analysed, with three technical replicates performed for each biological replicate. The exact number of biological replicates (*n*) is indicated in the corresponding figure description and/or table title. The mean value of the three technical replicates was used for each biological replicate.

### 4.2. Determination of Photosynthetic Pigments in the Pitcher Tissue

#### 4.2.1. Chlorophyll and Carotenoid Contents in the Glandular Zone of Pitcher Traps

Chlorophyll and carotenoid contents in the glandular zone tissue of pitcher traps were determined spectrophotometrically according to Sims and Gamon [76], with modifications. Tissue samples (0.05 g) were homogenised in mortar with 2 mL of cold acetone/Tris-HCl buffer (80:20, *v*/*v*; 0.1 mM Tris-HCl, pH 7.8). The homogenates were centrifuged at 10,000× *g* for 5 min at 4 °C. Subsequently, 1 mL of the supernatant was transferred to a new tube and diluted with 1 mL of the extraction mixture. Absorbance was measured at 537, 647, 663, and 470 nm. The extraction mixture without plant material was used as a blank.

The concentrations of chlorophyll *a* (Chl *a*), chlorophyll *b* (Chl *b*), the anthocyanin correction term, and total carotenoids were calculated using the following equations:

$$Chl\;a=0.01373A_{663}-0.000897A_{537}-0.003046A_{647}$$

$$\begin{array}{c} Chl\;b=0.02405A_{647}-0.004305A_{537}-0.005507A_{663}\\ Anthocyanins=0.08173A_{537}-0.00697A_{647}-0.002228A_{663} \end{array}$$

$$Carotenoids=\frac{A_{470}-\left[17.1(Chl\;a+Chl\;b)-9.479(Anthocyanin)\right]}{119.26}$$

The pigment concentrations obtained from the equations were corrected for the 4-fold dilution and the volume of the extraction mixture and expressed as µmol g^−1^ FW.

#### 4.2.2. Anthocyanin Content in the Glandular Zone of Pitcher Traps

Anthocyanin content in the tissue of glandular zone of pitcher traps was determined spectrophotometrically according to Sims and Gamon [76], with modifications. Tissue samples (0.05 g) were homogenised in 2 mL of a cold mixture of methanol, water, and hydrochloric acid (90:9:1; *v*/*v*/*v*). The homogenates were centrifuged at 9000× *g* for 5 min at room temperature (RT). Next, 1 mL of the supernatant was transferred to a new tube and diluted with 1 mL of the extraction mixture. Absorbance was measured at 529 and 650 nm. The extraction mixture without plant material was used as a blank.

To correct for the contribution of chlorophyll degradation products to absorbance at 529 nm, absorbance was calculated according to the following equation:

$$AA=A_{529}-(0.288A_{650})$$

AA represents the corrected anthocyanin absorbance. Total anthocyanin content was calculated using a molar absorption coefficient of 30,000 L mol^−1^ cm^−1^ at 529 nm, corrected for the 4-fold dilution and extraction volume, and expressed as µmol g^−1^ FW.

### 4.3. Measurement of Hydroxyl Radical (^•^OH) Level in the Digestive Fluid

The level of ^•^OH in the digestive fluid was determined colorimetrically using a modified Griess assay with 4-nitro-1H-imidazole, according to Deng et al. [77], with modifications.

To 1.5 mL of the digestive fluid, 3 µL of 50% TCA was added. In the blank sample, the digestive fluid was replaced with distilled water. The samples were centrifuged at 7000× *g* for 10 min at 4 °C, and the deproteinised supernatant was collected. Then, 5 µL of 100 mM 4-nitro-1H-imidazole, prepared in 2 M NaOH, was added to 1 mL of deproteinised supernatant and incubated for 15 min at RT. Next, 100 µL of the reaction mixture was combined with 100 µL of Griess reagent (Sigma G4410, St. Louis, MO, USA). The samples were incubated for 15 min at room temperature in the dark, and absorbance was measured at 540 nm using a microplate reader (CLARIO star plus, BMG LABTECH, Ortenberg, Germany). In parallel, nitrite ions naturally present in the sample were measured in samples without the addition of 4-nitro-1H-imidazole.

Hydroxyl radical (^•^OH) levels were expressed as the amount of NO_2_^−^ released from 4-nitro-1H-imidazole, calculated from a NaNO_2_ standard curve and expressed per trap (nmol NO_2_^−^ trap^−1^).

### 4.4. Measurement of H_2_O_2_ Content in the Digestive Fluid

Content of H_2_O_2_ was measured using the KI-based colorimetric method, according to Junglee et al. [78], with modification by Wal et al. [3]. The digestive fluid (700 µL) was combined with 50 µL of 1% TCA and 250 µL of 2 M KI in 10 mM potassium phosphate buffer, pH 7.0. Samples were incubated for 15 min at RT in darkness, and absorbance was measured at 390 nm. Distilled water was used instead of the digestive fluid in the blank. H_2_O_2_ content was estimated from a calibration curve prepared with H_2_O_2_ and expressed as pmol H_2_O_2_ trap^−1^.

### 4.5. Measurement of POx Activity in the Digestive Fluid

Activity of POx in the digestive fluid was determined spectrophotometrically based on the oxidation of pyrogallol to purpurogallin, according to Saunders et al. [79] with modification by Wal et al. [3]. The digestive fluid 0.9 mL was buffered with 0.1 mL of a mixture containing 1 M K-phosphate buffer, pH 7.0, 30 mM EDTA, 10 mM DTT, and 10 mM PMSF. Then, 300 µL of the buffered digestive fluid was combined with 2.5 mL of 7 mM pyrogallol prepared in 50 mM K-phosphate buffer, pH 7.0. The reaction was initiated by adding 20 µL of H_2_O_2_ to 280 µL of the reaction mixture. In the blank, H_2_O_2_ was replaced with distilled water. The formation of purpurogallin was monitored at 430 nm. POx activity was expressed as nmol purpurogallin min^−1^ trap^−1^.

### 4.6. Measurement of SOD Activity in the Digestive Fluid

Activity of SOD was determined spectrophotometrically according to Alam and Kataria [80], with modifications. The enzymatic extract prepared from proteins precipitated from the digestive fluid was used in the assay. For this purpose, a 5-fold volume of 80% ammonium acetate solution was added to the entire volume of the digestive fluid, and the samples were incubated overnight at −20 °C. Then, the samples were centrifuged for 20 min at 12,000× *g* at 4 °C. The supernatant was removed, and the obtained pellet was briefly dried and dissolved in 200 µL of 0.1 M K-phosphate buffer, pH 7.8.

The reaction mixture contained 200 µL of the enzyme extract prepared as described above, 100 µL of 0.19 M methionine, 100 µL of 11 mM riboflavin, and 50 µL of 1.7 mM nitroblue tetrazolium chloride (NBT). A zero sample corresponding to 100% reduction of NBT to formazan was prepared in the same way, but the enzyme extract was replaced with 200 µL of 0.1 M K-phosphate buffer, pH 7.8.

The reaction mixtures were exposed to light for 10 min. After incubation, the absorbance of the formed formazan was measured at 560 nm using a microplate reader. One unit of SOD activity was defined as the amount of the enzyme extract causing 50% inhibition of formazan formation compared with the zero sample. SOD activity was expressed as U min^−1^ trap^−1^.

### 4.7. Measurement of PAO and DAO Activity in the Digestive Fluid

The activities of PAO and DAO in the digestive fluid were determined spectrophotometrically using a horseradish peroxidase (HRP)-coupled reaction. The assay was performed according to the methods described by Delis et al. [81] and Krasuska et al. [82], with modifications.

For PAO activity determination, the reaction mixture contained 50 mM potassium phosphate buffer, pH 6.5, 10 mM pyrogallol, 1 U horseradish peroxidase (HRP), and 10 mM spermine. The buffered digestive fluid, pH 6.5, was added to the reaction mixture in a volume of 630 µL.

For DAO activity determination, 2.5 mM putrescine was used as the substrate, and 865 µL of the buffered digestive fluid (pH 6.5) was added to the reaction mixture.

At the same time, blanks were prepared. The first blank contained all components of the reaction mixture and the digestive fluid, but without the substrate. The second blank contained the substrate, but the digestive fluid was replaced with buffer.

Immediately after adding the digestive fluid to the reaction mixture, absorbance was measured at 420 nm for 30 min using a microplate reader. The reaction rate was calculated from the change in absorbance over time, after correcting for both blank samples. The results were expressed as nmol H_2_O_2_ min^−1^ trap^−1^.

### 4.8. Histochemical Localisation of Phenolic Compounds (PCs) in the Tissue of the Trap

Histochemical localisation of phenolic compounds (PCs) was performed according to Reeve [83] and Staszek et al. [84]. Rectangular fragments of the tissue were excised from the lower part of the pitchers and placed, four fragments per Petri dish. Reagent mixture consisted of equal volumes of 10% sodium nitrite (NaNO_2_), 20% urea (CH_4_N_2_O), and 10% acetic acid (CH_3_COOH). The tissue fragments were immersed in the mixture for 4 min in the dark. After incubation, the staining reaction was stopped by adding two volumes of 2 M NaOH. Control samples were prepared by replacing acetic acid with NaOH, and the solution used to stop the reaction was replaced with distilled water.

After staining, the tissue fragments were photographed using a stereoscopic microscope equipped with an integrated Nikon H550S (Nikon Corporation, Tokyo, Japan) camera. The brown colouration indicated the presence of PCs.

### 4.9. Measurement of the Flavonoid Level in the Digestive Fluid

The total flavonoid content in the digestive fluid was determined according to the method described by Wal et al. [3]. The digestive fluid (1.5 mL) was mixed with 75 µL of 5% NaNO_2_ solution, vortexed, and incubated for 5 min at 22 °C. Then, 150 µL of 10% aluminium chloride solution was added, and the reaction mixture was mixed and incubated for 6 min at 22 °C. After incubation, 500 µL of 1 M NaOH was added and mixed thoroughly. The absorbance was measured at 496 nm. The blank was prepared by replacing the digestive fluid with distilled water. Flavonoid content was calculated using a standard curve prepared with quercetin and expressed as µg quercetin trap^−1^.

### 4.10. Flavonoids Localisation in the Glandular Zone of Pitcher Traps

Flavonoids localisation was performed using 2-aminoethyl diphenylborinate (DPBA), according to the method described by Staszek et al. [84], with modifications. Hand-cut cross sections of the glandular zone and epidermal peels from the inner lower surface of the pitchers were immediately immersed in an aqueous 0.01 mM DPBA solution containing 0.01% (*v*/*v*) Triton X-100. The tissue fragments were incubated for 10 min at RT in the dark. After staining, the samples were washed with distilled water and mounted in distilled water for microscopic observation.

Fluorescence imaging was performed using an FV-500 Olympus confocal laser scanning microscope (Olympus Corporation, Tokyo, Japan) in sequential scanning mode with 20× and 40× objectives. The following excitation/emission settings were used: 405 nm/430–460 nm, 488 nm/505–525 nm, and 543 nm/560 IF. Confocal microscopy images were assembled into figures using Adobe Photoshop CS6, version 13.0 (Adobe Systems Incorporated, San Jose, CA, USA); original microphotographs are attached as Supplementary Materials (Figures S1–S20).

### 4.11. Measurement of Reducing Capacity in the Digestive Fluid

The reducing capacity of the digestive fluid was determined according to Ferreira et al. [85], with modifications. The digestive fluid was buffered with 2 M sodium phosphate buffer (pH 6.6) at a 1:9 ratio. Then, an equal volume of 1% potassium ferricyanide solution was added to the buffered sample. The reaction mixture was incubated for 20 min at 50 °C in the dark. After incubation, 250 µL of 100% TCA was added, and the samples were kept for 5 min at RT in the dark. The mixtures were then centrifuged at 15,000× *g* for 15 min. Next, 1 mL of 0.1% ferric chloride was added to 2.5 mL of the obtained supernatant and incubated for 10 min at RT. Absorbance was measured at 700 nm using a microplate reader. The results were presented as % reducing capacity relative to the 0.125 mM ascorbic acid solution, which accounted for 100%.

### 4.12. Measurement of the Total Content of Compounds with Sulfhydryl Groups (-SH) in the Digestive Fluid

The content of compounds containing free sulfhydryl groups (-SH) in the digestive fluid was determined using 5,5′-dithiobis(2-nitrobenzoic acid) (DTNB), according to Wal et al. [3], with modifications.

For the determination of total free -SH groups, the digestive fluid was buffered with 1 M phosphate buffer, pH 7.5. Then, phenylmethylsulfonyl fluoride (PMSF) and EDTA were added to the sample. The buffered digestive fluid was mixed with 6 mM DTNB prepared in 0.1 M phosphate buffer, pH 7.5. The reaction mixture was incubated for 10 min at RT in the dark, and absorbance was measured at 420 nm.

At the same time, the content of low-molecular-weight thiol compounds was determined after protein precipitation with TCA. For this purpose, 3 µL of 100% TCA was added to 500 µL of the digestive fluid, and the mixture was incubated for 10 min at RT. The samples were then centrifuged for 10 min at 20,071× *g* at 4 °C. After centrifugation, the clear supernatant was collected for further analysis. The next steps in the procedure were carried out as described above. The concentration of 2-nitro-5-thiobenzoate (TNB) was calculated using the extinction coefficient ε = 14.15 mM^−1^ cm^−1^. The results were expressed as µmol TNB trap^−1^.

### 4.13. Statistical Analysis

Data were analysed using Statistica software. Results are presented as means ± SD. Differences between experimental treatments were evaluated using one-way ANOVA followed by Fisher’s LSD post hoc test, at *p* ≤ 0.05.

## 5. Conclusions

The development of the jug-shaped trap of *Nepenthes* requires coordinated action of many regulators, with ROS being crucial among them. ROS have a dual role; thus, regulation of their content requires activation of a precise modulatory system, consisting of enzymes and non-enzymatic compounds. The initial phases of *N. × ventrata* trap development depend on the formation of O_2_^•−^ and ^•^OH in the fluid. We propose that to gain a high level of these ROS, pro-oxidative activity of POx is stimulated. On the contrary, the final stages of trap life are accompanied by an increased H_2_O_2_ level. Increased H_2_O_2_ is linked to high SOD activity, and elevated PAO and DAO activities point to the involvement of bioactive amine catabolism in H_2_O_2_ generation. Maturation of the trap is the stage of pigmentation and the release of PCs and flavonoids into the fluid. These non-enzymatic antioxidants, in addition to their role in prey attraction, are also helpful in the modulation of ROS levels. In the mature trap, ROS scavenging may operate as a protective mechanism against oxidation of digestive enzymes in the digestive fluid. Thus, due to ROS presence and action, the trap is ready to fulfil its physiological functions. Future studies should focus on the mechanisms by which ROS regulation supports the pitcher development and digestive function in *Nepenthes*.

## Figures and Tables

**Figure 1:**
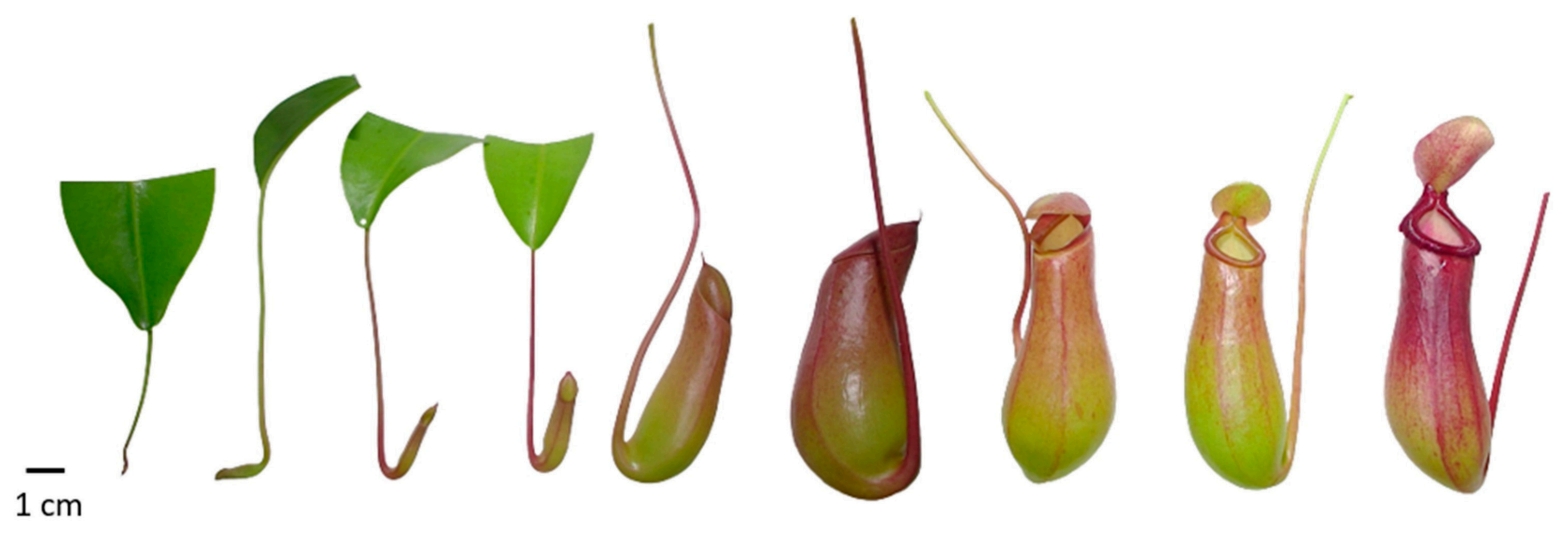
Schematic representation of *N. × ventrata* pitcher development from a small structure at the end of the tendril to a fully mature, opened trap (photograph by M. Piekarniak).

**Figure 2:**
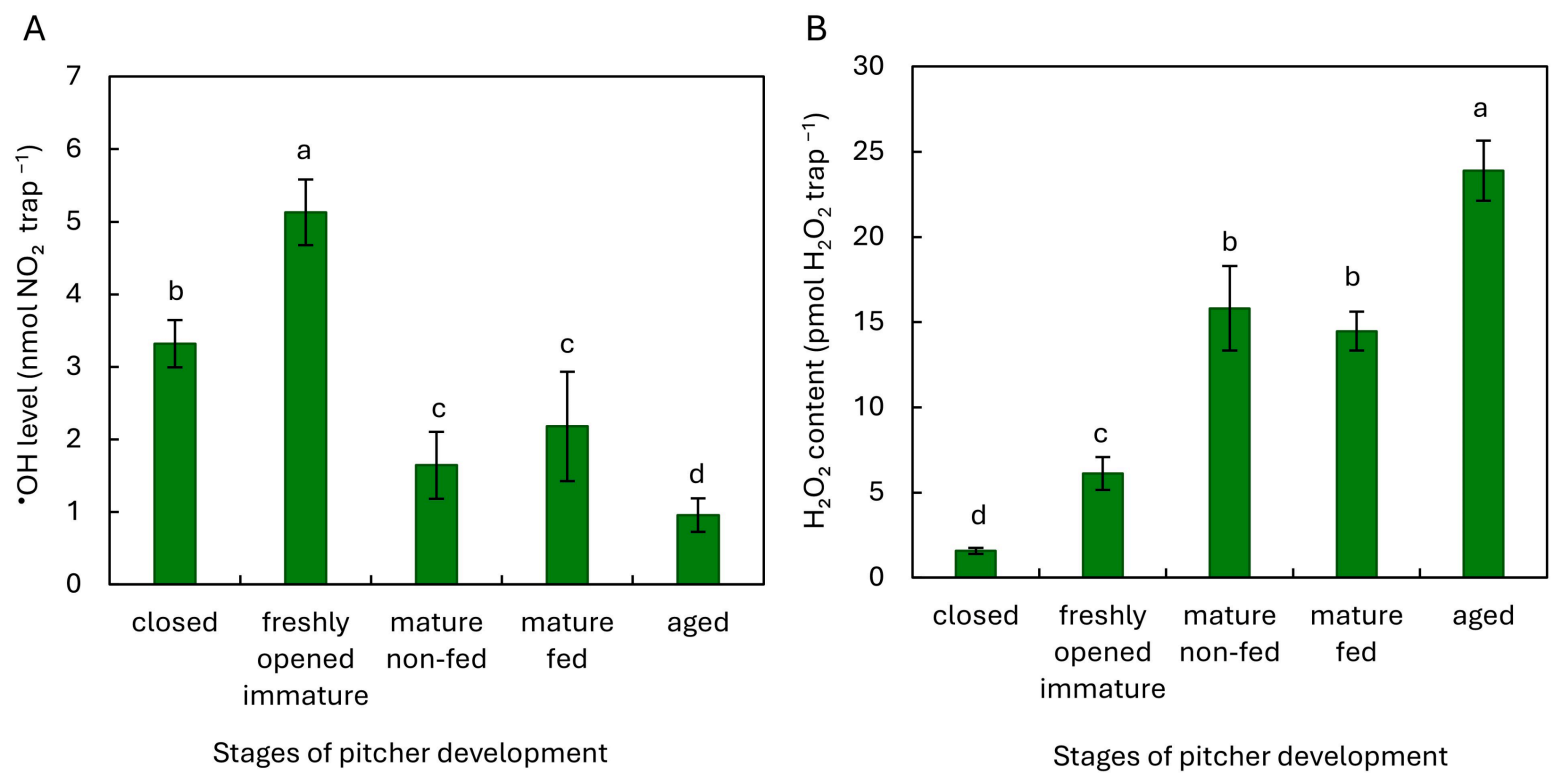
Concentration of hydroxyl radical (^•^OH) (**A**) and hydrogen peroxide (H_2_O_2_) (**B**) in the digestive fluid of pitchers at different developmental stages: closed, freshly opened immature, mature non-fed, mature fed, and pitchers showing signs of senescence (aged). Values are means ± SD, *n* = 4. Letters (a–d) indicate significant differences between developmental stages according to Fisher’s LSD post hoc test at *p* ≤ 0.05.

**Figure 3:**
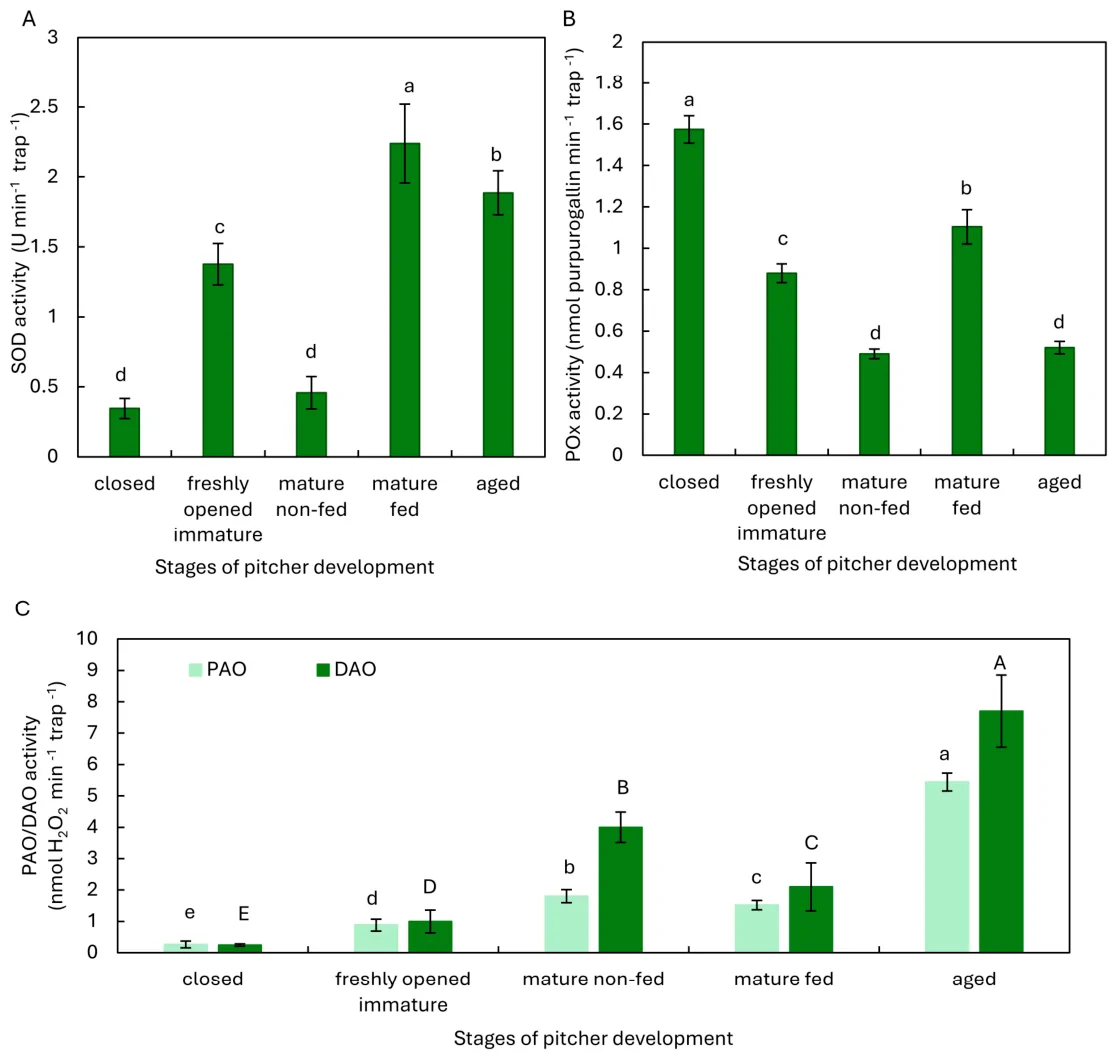
The activity of superoxide dismutase (SOD) (**A**), peroxidase (POx) (**B**), and polyamine oxidase (PAO) and diamine oxidase (DAO) (**C**) in the digestive fluid of pitchers at different developmental stages: closed, freshly opened immature, mature non-fed, mature fed, and pitchers showing signs of senescence (aged). Values are means ± SD, *n* = 5. For SOD (**A**) and POx (**B**), activity letters (a–d) indicate significant differences between developmental stages according to Fisher’s LSD post hoc test at *p* ≤ 0.05. For PAO and DAO activity (**C**), lowercase letters (a–e) indicate significant differences for PAO activity, whereas uppercase letters (A–E) indicate significant differences for DAO activity, between developmental stages, according to Fisher’s LSD post hoc test at *p* ≤ 0.05.

**Figure 4:**
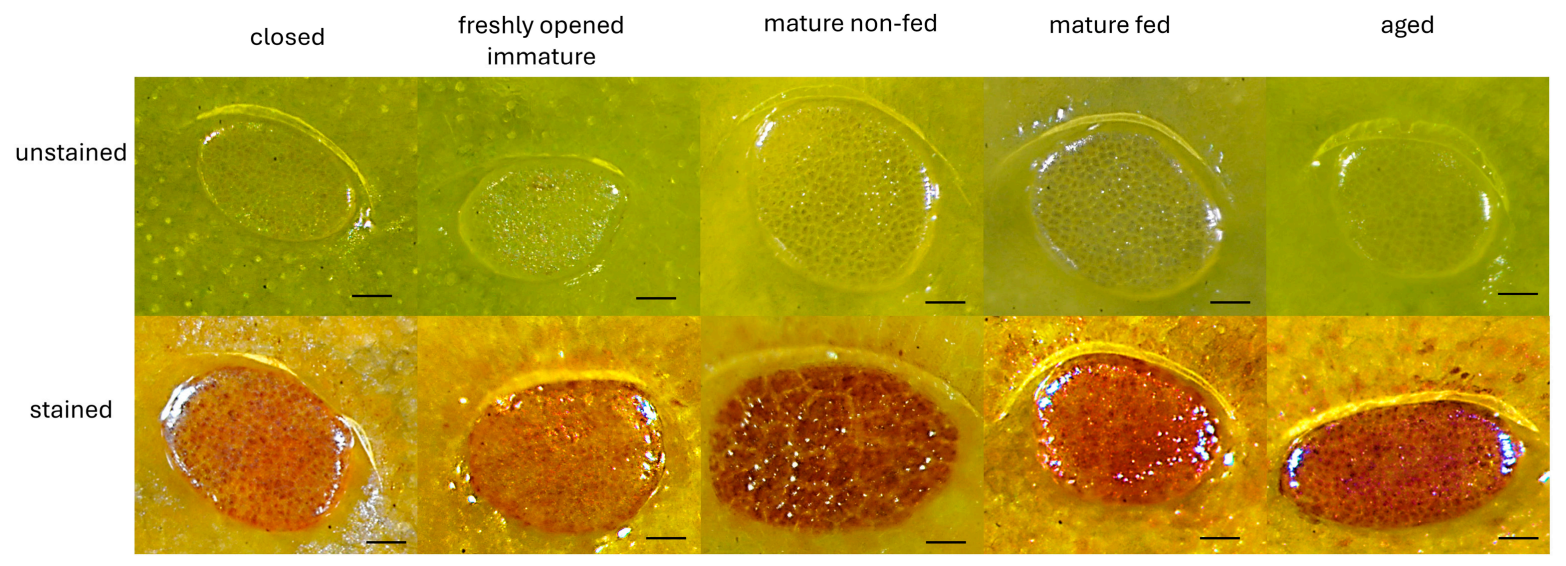
Histochemical localisation of phenolic compounds (PCs) in the glandular zone of pitchers at different developmental stages: closed immature, freshly opened immature, mature opened non-fed, mature opened fed, and pitchers with visible symptoms of senescence (aged). Unstained samples are shown in the upper row, and stained samples are shown in the lower row. Representative images are presented. Scale bars = 100 µm.

**Figure 5:**
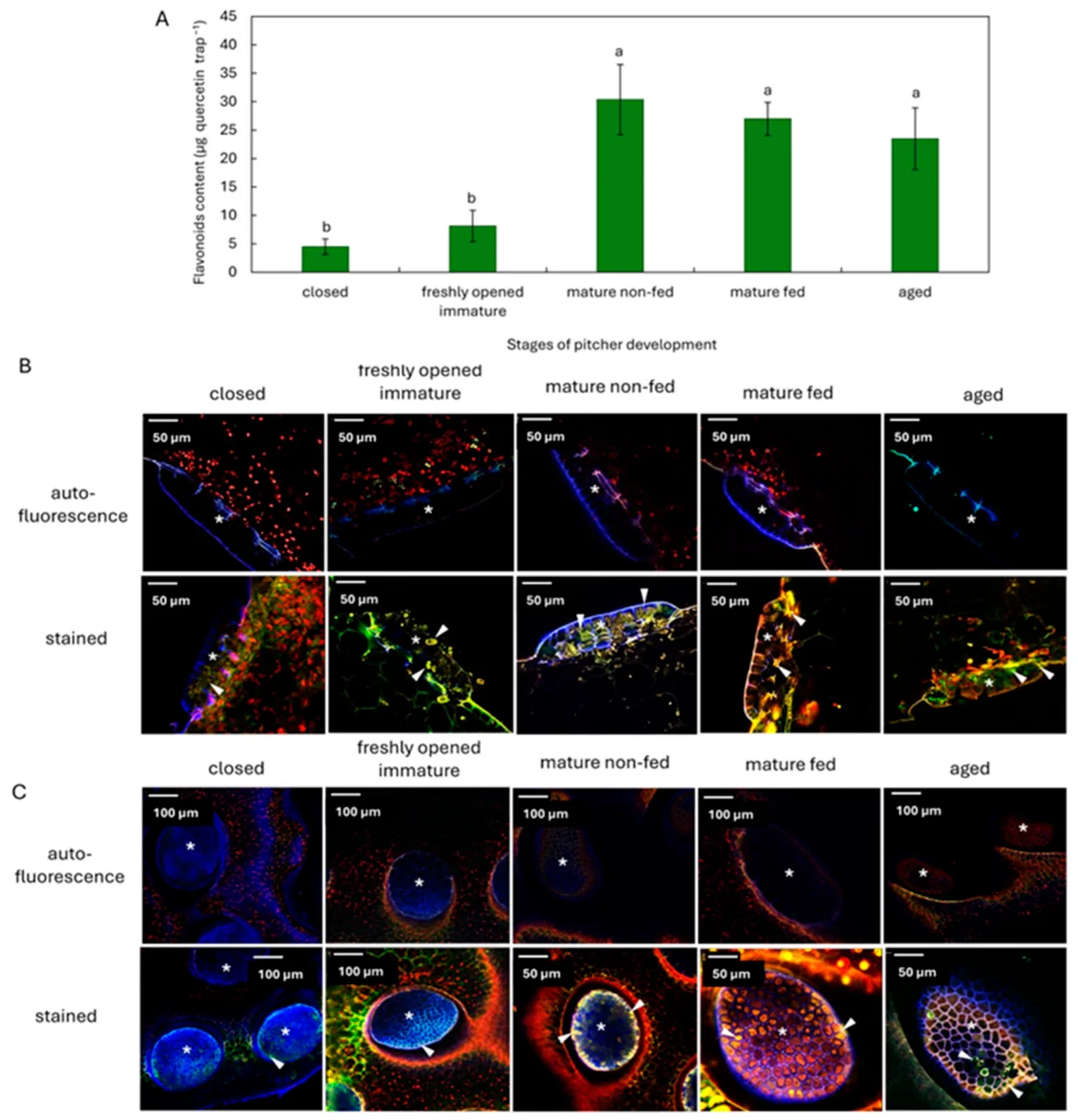
The flavonoid content (**A**) in the digestive fluid of pitchers at different developmental stages: closed, freshly opened immature, mature non-fed, mature fed, and pitchers showing signs of senescence (aged). Values are means ± SD, *n* = 5. Letters (a, b) indicate significant differences between developmental stages according to Fisher’s LSD post hoc test at *p* ≤ 0.05. Histochemical localisation of flavonoids by DPBA-stained fluorescence in the glandular zone of pitcher traps at different developmental stages: cross sections of the glandular zone (**B**) and epidermal peels from the glandular tissues (**C**). In each panel set, the upper row shows unstained autofluorescence of tissues, and the lower row shows the corresponding DPBA-stained samples from: closed immature, opened immature, mature opened non-fed, mature opened fed, and pitchers with visible symptoms of senescence. DPBA-stained flavonoids are visible as yellow and green fluorescence, and arrows indicate examples of flavonoid localisation. Asterisks mark the central part of the glands. Representative confocal images are shown.

**Figure 6:**
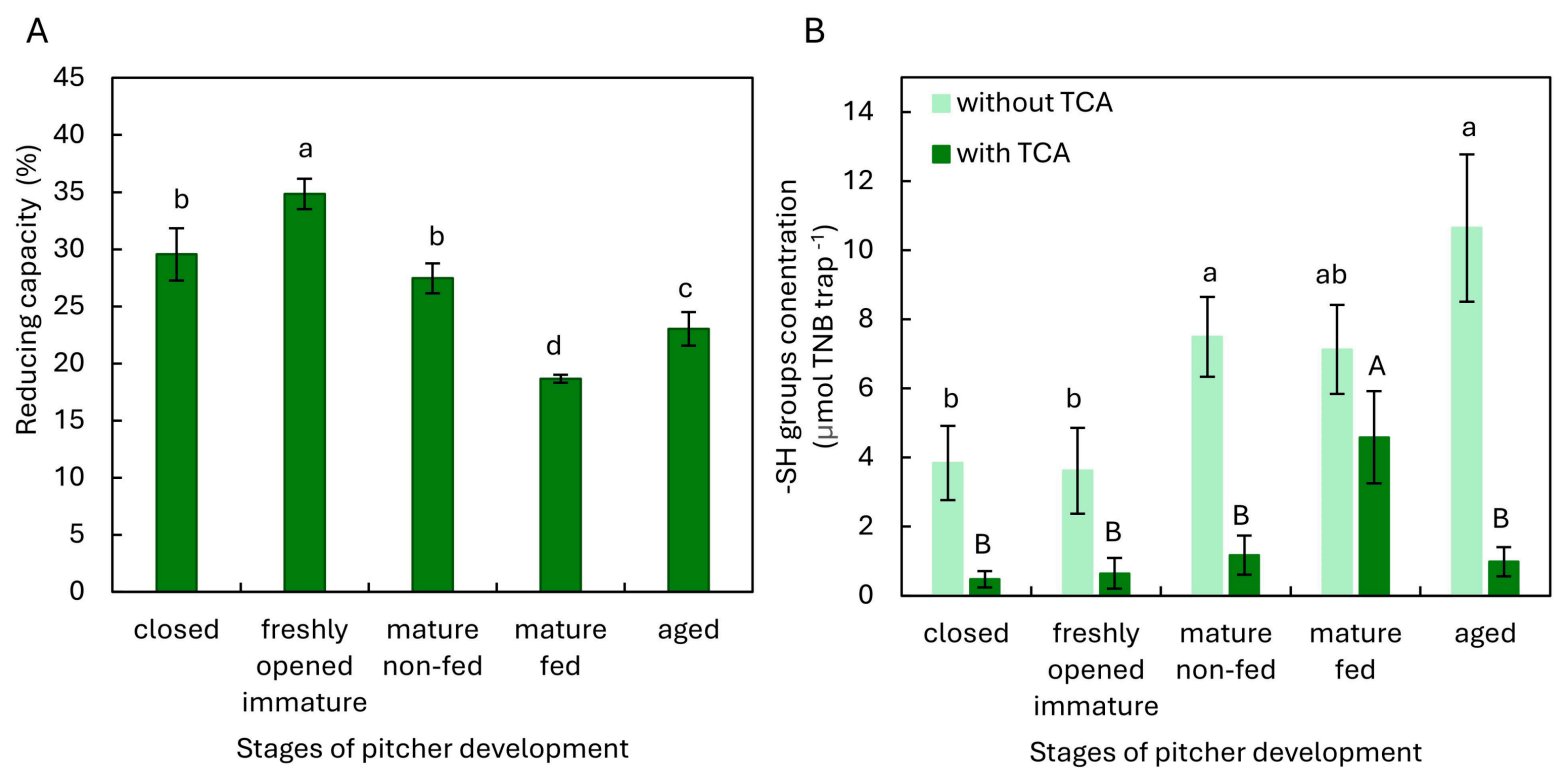
Reducing capacity (**A**) and the concentration of compounds containing sulfhydryl groups (-SH) (**B**) in the digestive fluid of pitchers at different developmental stages: closed, freshly opened immature, mature non-fed, mature fed, and pitchers showing signs of senescence (aged). Reducing capacity was expressed relative to that of a 0.125 mM ascorbic acid solution, which was defined as 100%. The assay of -SH groups was performed without TCA and after TCA protein precipitation. Values are means ± SD, *n* = 5. For data on reducing capacity, letters (a–d) indicate significant differences between developmental stages according to Fisher’s LSD post hoc test at *p* ≤ 0.05. For -SH groups, lowercase letters (a, b) indicate significant differences for samples without TCA, whereas uppercase letters (A, B) indicate significant differences for samples with TCA, differences between developmental stages according to Fisher’s LSD post hoc test at *p* ≤ 0.05.

**Figure 7:**
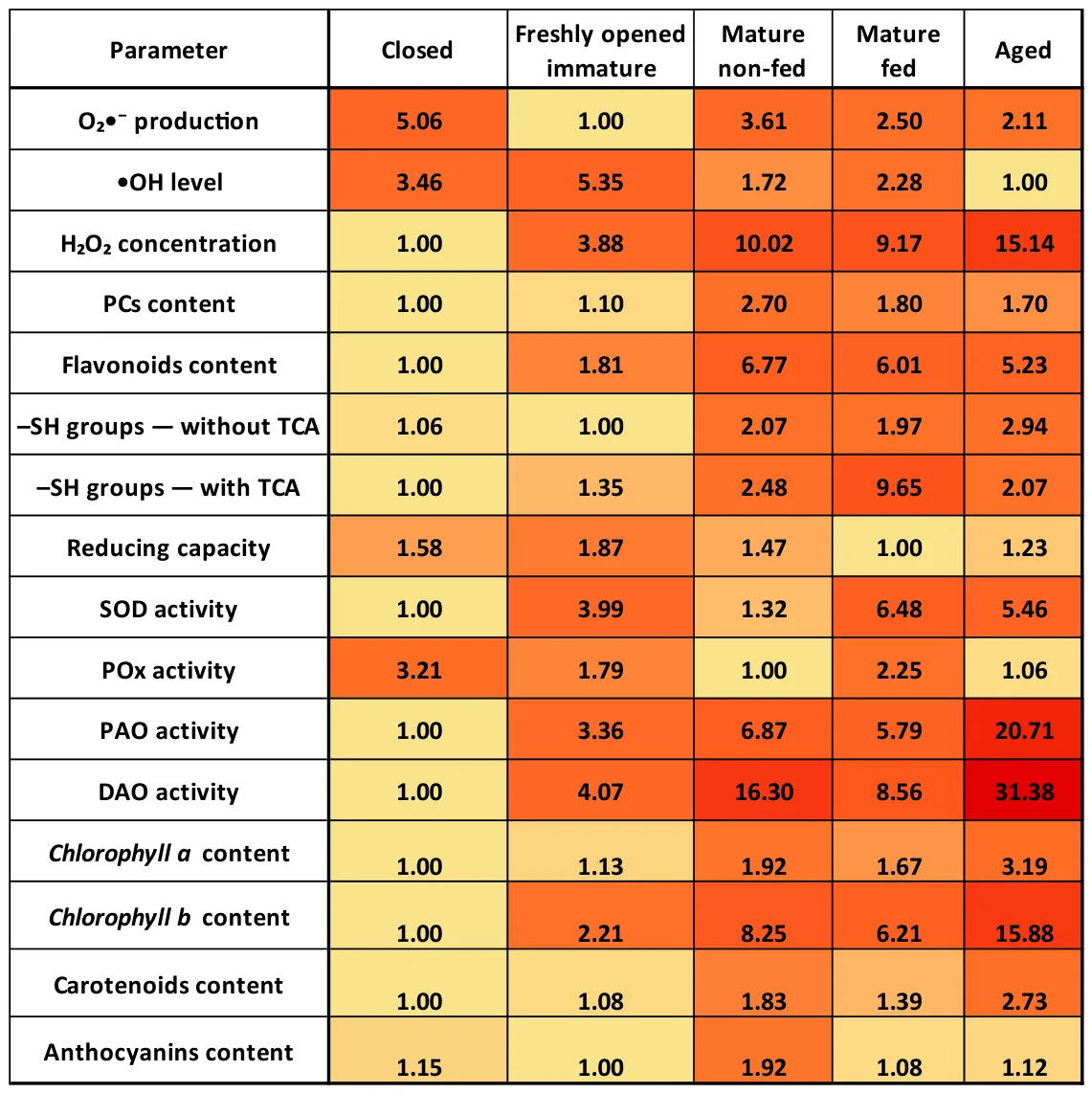
Heat map summarising relative changes in redox-related parameters, oxidoreductase activities, photosynthetic pigments and anthocyanin contents in *N*. *× ventrata* pitchers and digestive fluid at different developmental stages of the traps: closed, freshly opened immature, mature non-fed, mature fed, and aged. For each parameter, the lowest mean value across developmental stages was assigned as 1.00, and the remaining values were expressed as fold changes relative to this minimum. The colour gradient from light yellow to dark red indicates increasing relative values. All data presented in the heat map were obtained in the present study, except for O_2_^•−^ production and the content of phenolic compounds (PCs), which were previously published by Wal et al. [19], using the same experimental model of pitcher development as in the present study.

**Figure 8:**
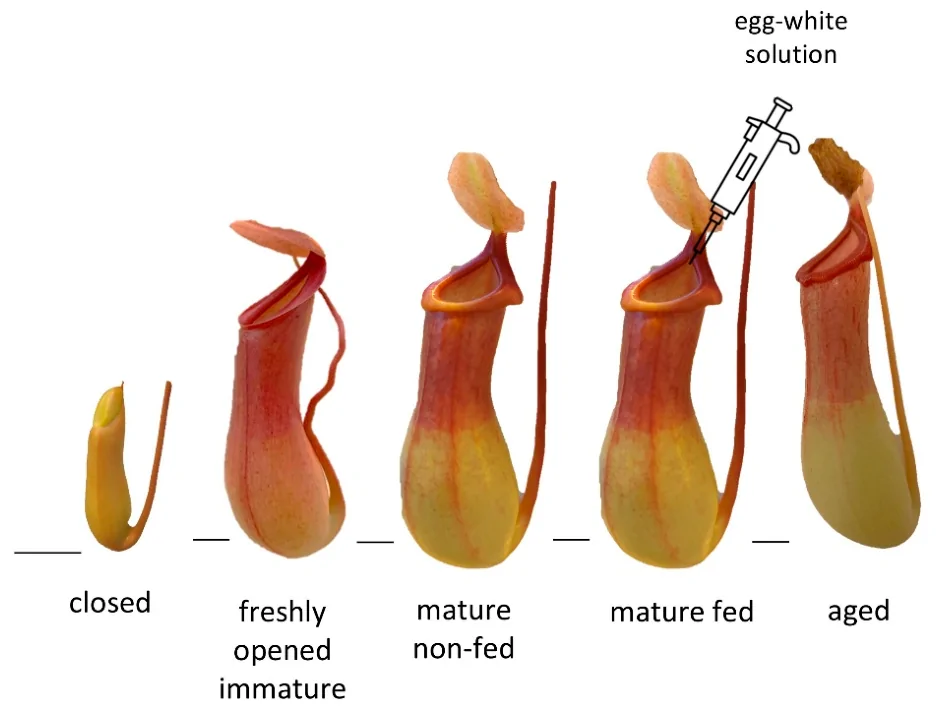
Pitchers of *N. × ventrata* at different developmental stages. From the left: a closed pitcher, a freshly opened immature pitcher, a mature non-fed pitcher, a mature fed pitcher, and an aged pitcher, with visible symptoms of senescence (drying of the lid). Scale bar = 1 cm.

**Table 1:** Chlorophyll *a*, chlorophyll *b*, carotenoid, and anthocyanin contents in the glandular zone of *N. × ventrata* pitcher traps at different developmental stages. Values are presented as means ± SD, *n* = 6. Different letters within each column indicate significant differences between developmental stages according to Fisher’s LSD post hoc test at *p* ≤ 0.05.

Developmental Stage of the Trap	Chlorophyll *a* Content (µmol g^−1^ FW)	Chlorophyll *b* Content (µmol g^−1^ FW)	Carotenoid Content (µmol g^−1^ FW)	Anthocyanin Content (µmol g^−1^ FW)
Closed	0.111 ± 0.037 d	0.024 ± 0.022 d	0.143 ± 0.029 c	0.160 ± 0.043 b
Freshly opened immature	0.125 ± 0.032 cd	0.053 ± 0.037 cd	0.155 ± 0.034 c	0.158 ± 0.023 b
Mature non-fed	0.213 ± 0.058 b	0.198 ± 0.037 b	0.262 ± 0.051 b	0.245 ± 0.034 a
Mature fed	0.185 ± 0.030 b	0.149 ± 0.036 b	0.199 ± 0.036 c	0.145 ± 0.029 b
Aged	0.354 ± 0.047 a	0.381 ± 0.108 a	0.391 ± 0.051 a	0.125 ± 0.054 b

## Data Availability

The data supporting the findings of this study are available from the corresponding author upon reasonable request. Supplementary Materials include additional microscopic images.

## Supplementary Materials

The following supporting information can be downloaded at: https://www.mdpi.com/article/10.3390/ijms27198462/s1.

## Abbreviations

The following abbreviations are used in this manuscript:

DAO	Diamine oxidase
PAO	Polyamine oxidase
PCs	Phenolic compounds
POx	Peroxidase
ROS	Reactive oxygen species
SOD	Superoxide dismutase
