# Supplementary material for: ROS Equilibrium in the Digestive Fluid of the Nepenthes × ventrata Trap at Various Developmental Stages
Source: Int J Mol Sci. 2026 Sep 23;27(19):8462. doi: 10.3390/ijms27198462 (PMC13664827; doi:10.3390/ijms27198462)
Supplement: Supplementary file 1 [file ijms-27-08462-s001.zip › ijms-4517795-supplementary.pdf]

Supplementary information:

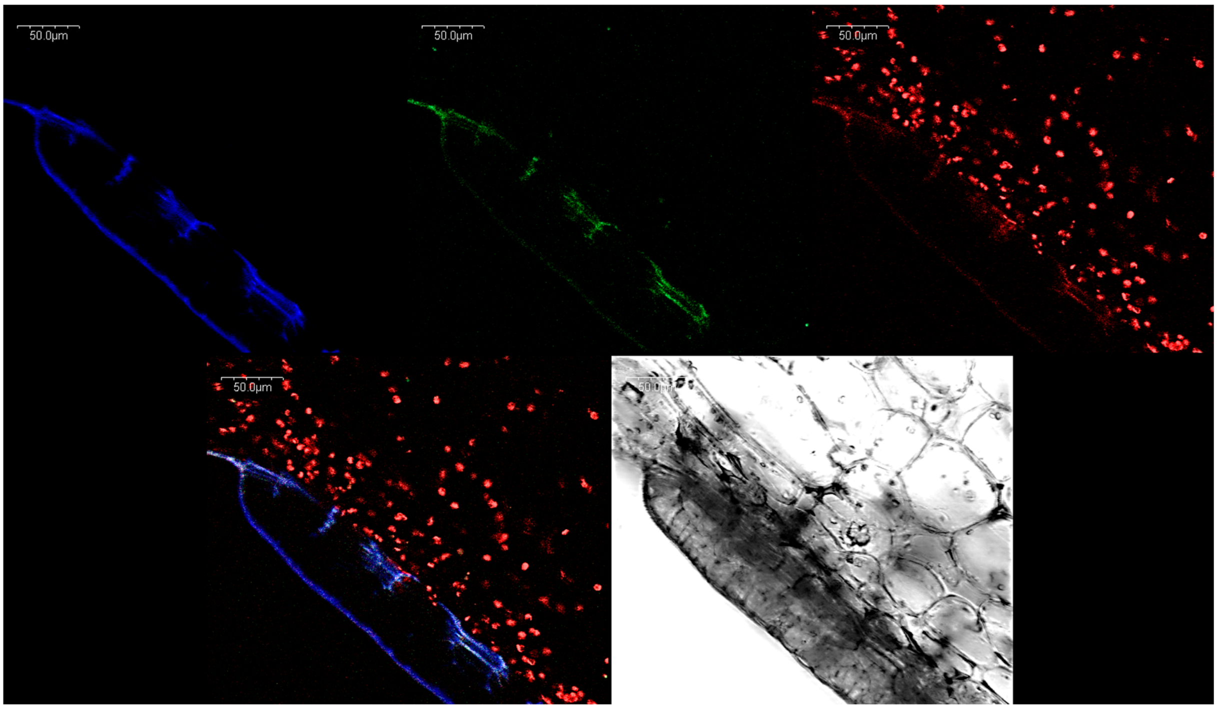

Figure S1

Autofluorescence of unstained cross-sections from the glandular zone of closed pitchers.

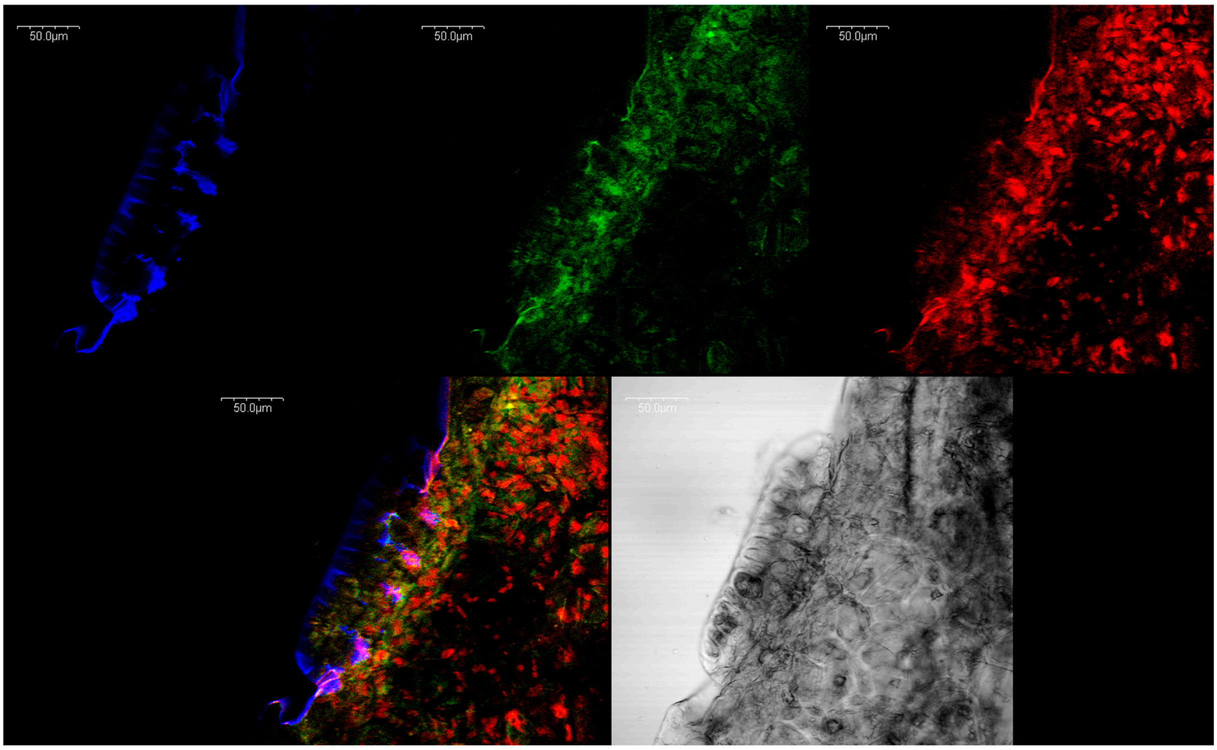

Figure S2

Fluorescence of DPBA-stained cross-sections from the glandular zone of closed pitchers.

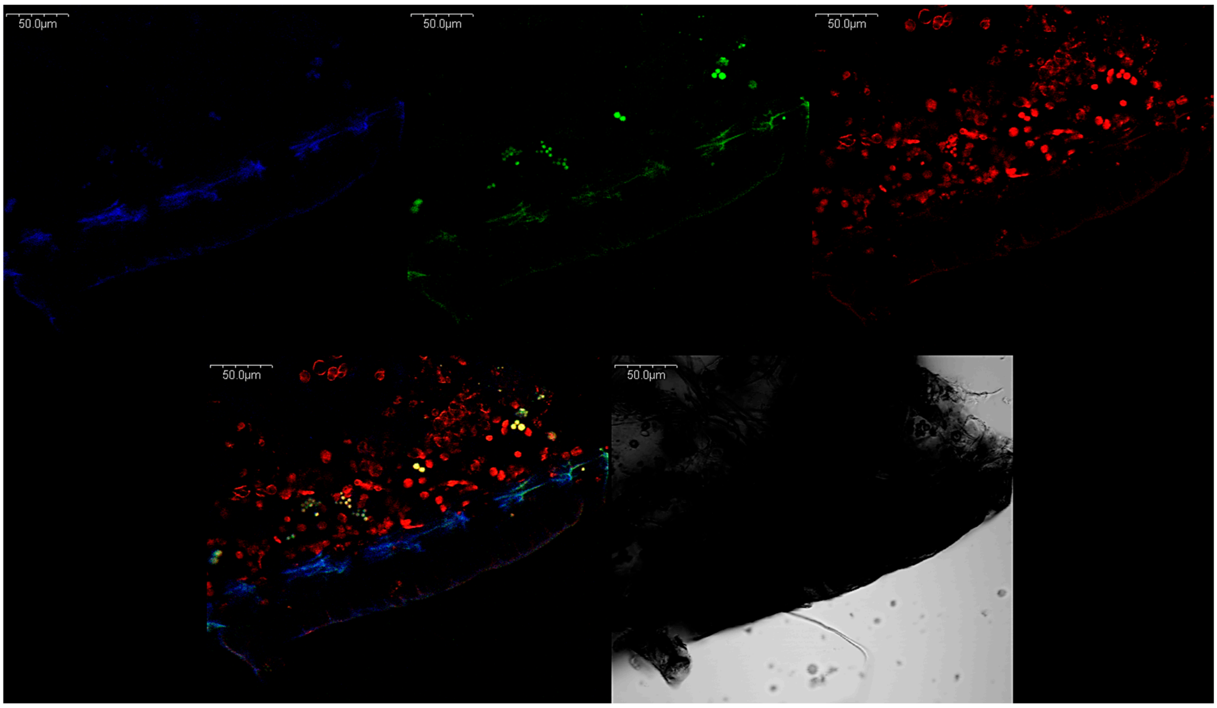

Figure S3

Autofluorescence of unstained cross-sections from the glandular zone of freshly opened immature pitchers.

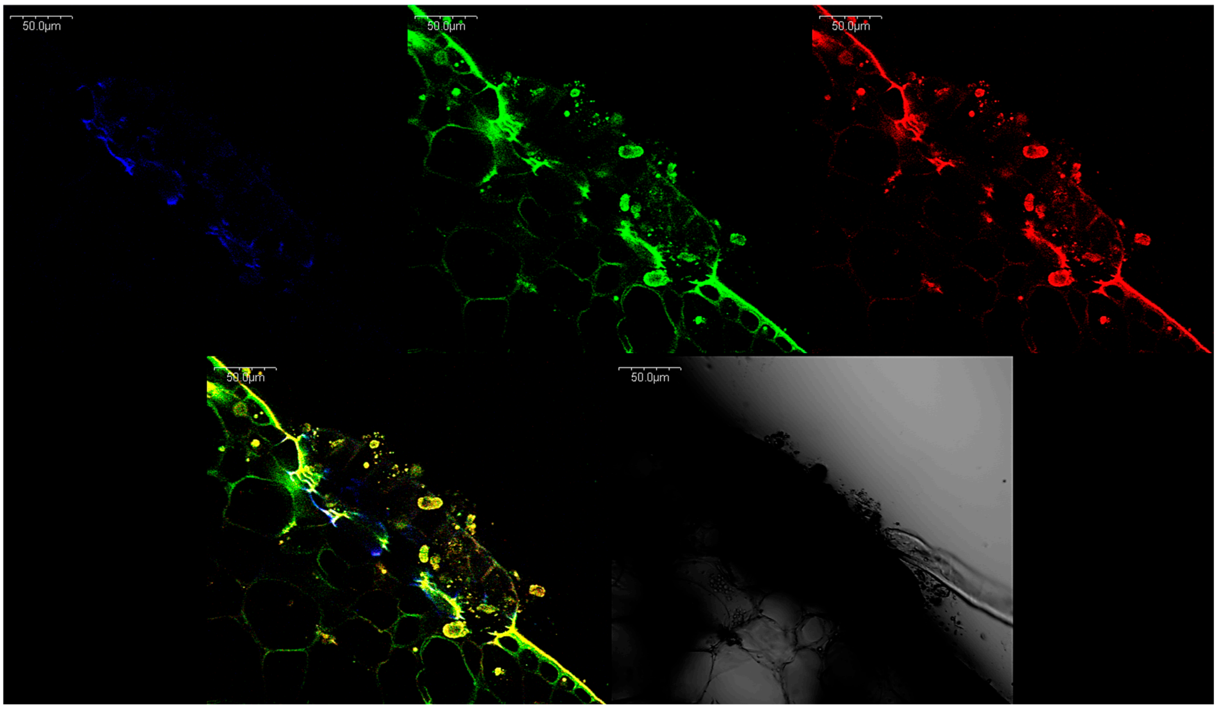

Figure S4

Fluorescence of DPBA-stained cross-sections from the glandular zone of freshly opened immature pitchers.

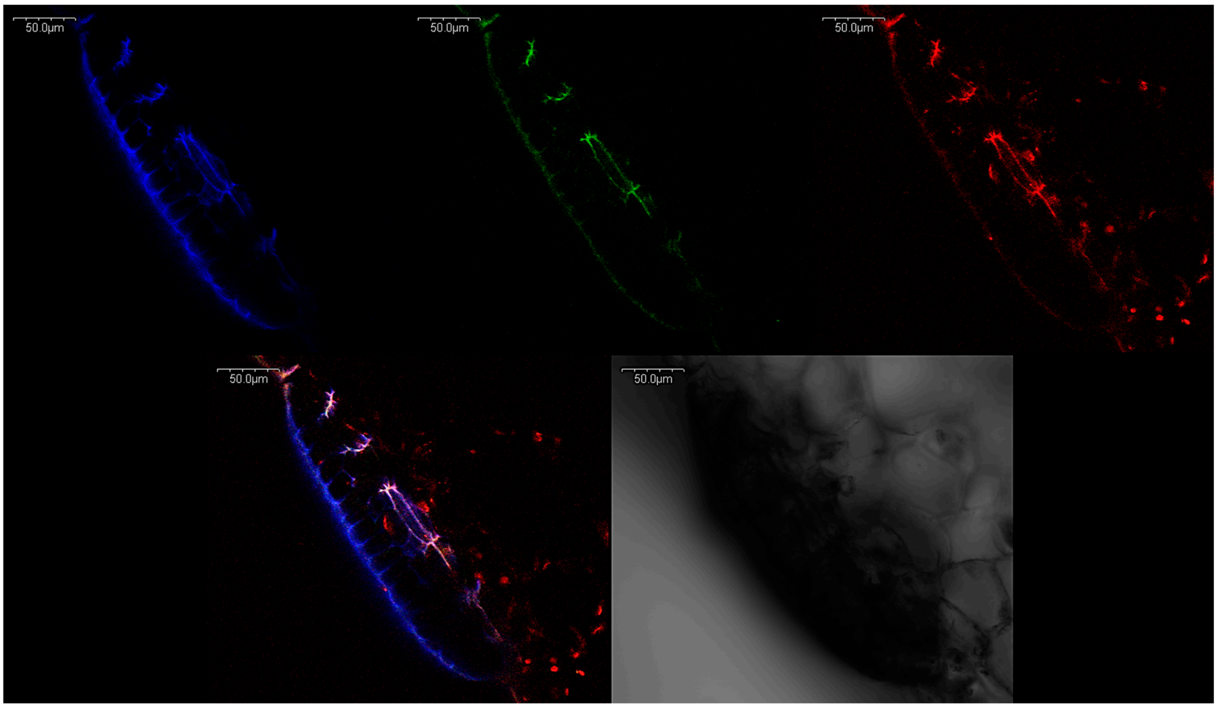

Figure S5

Autofluorescence of unstained cross-sections from the glandular zone of mature non-fed pitchers.

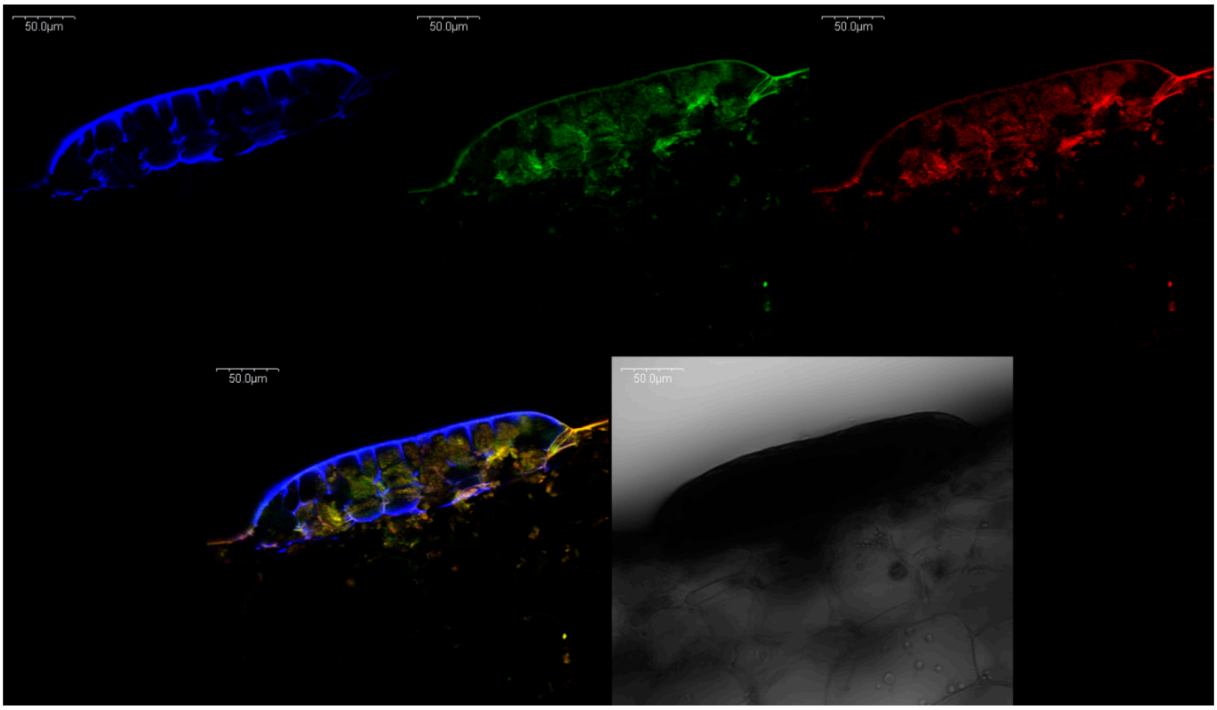

Figure S6

Fluorescence of DPBA-stained cross-sections from the glandular zone of mature non-fed pitchers.

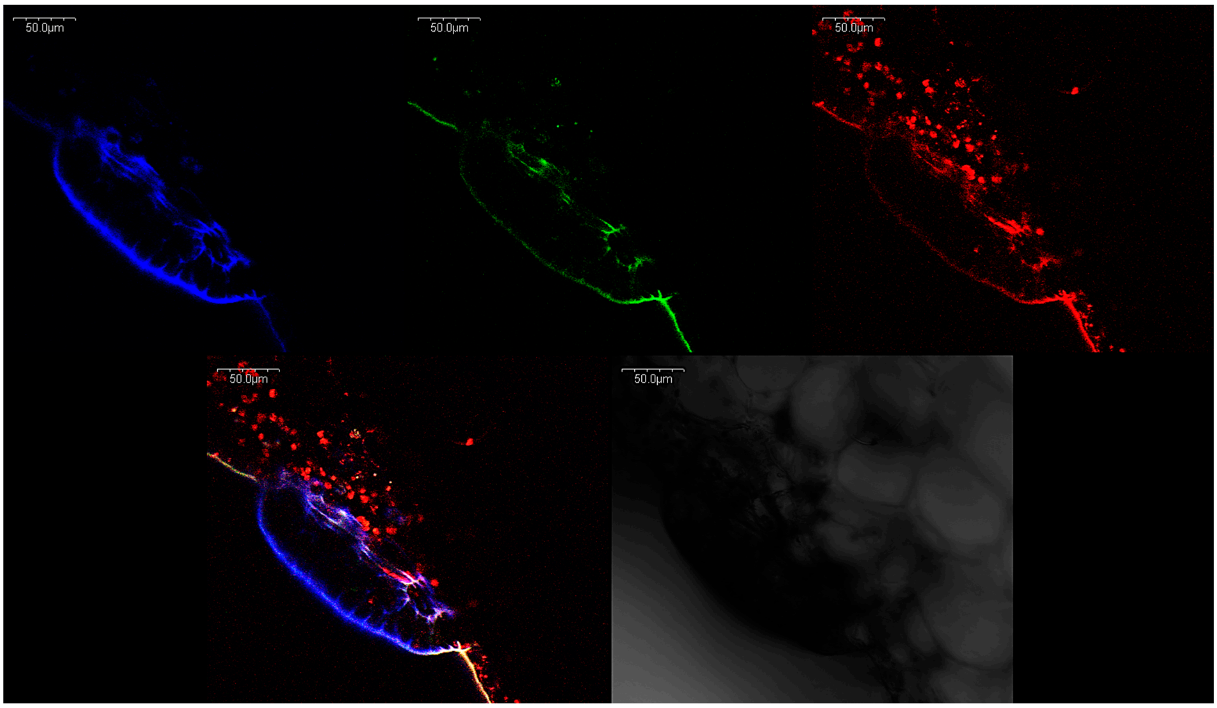

Figure S7

Autofluorescence of unstained cross-sections from the glandular zone of mature fed pitchers.

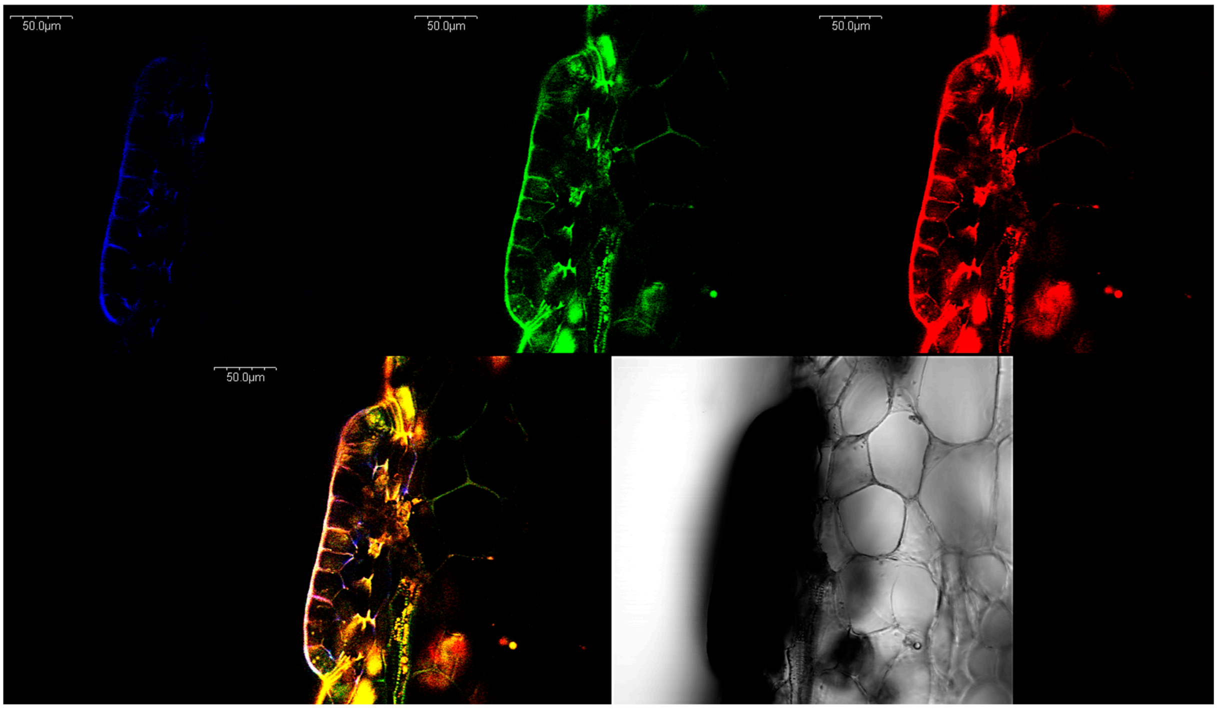

Figure S8

Fluorescence of DPBA-stained cross-sections from the glandular zone of mature fed pitchers.

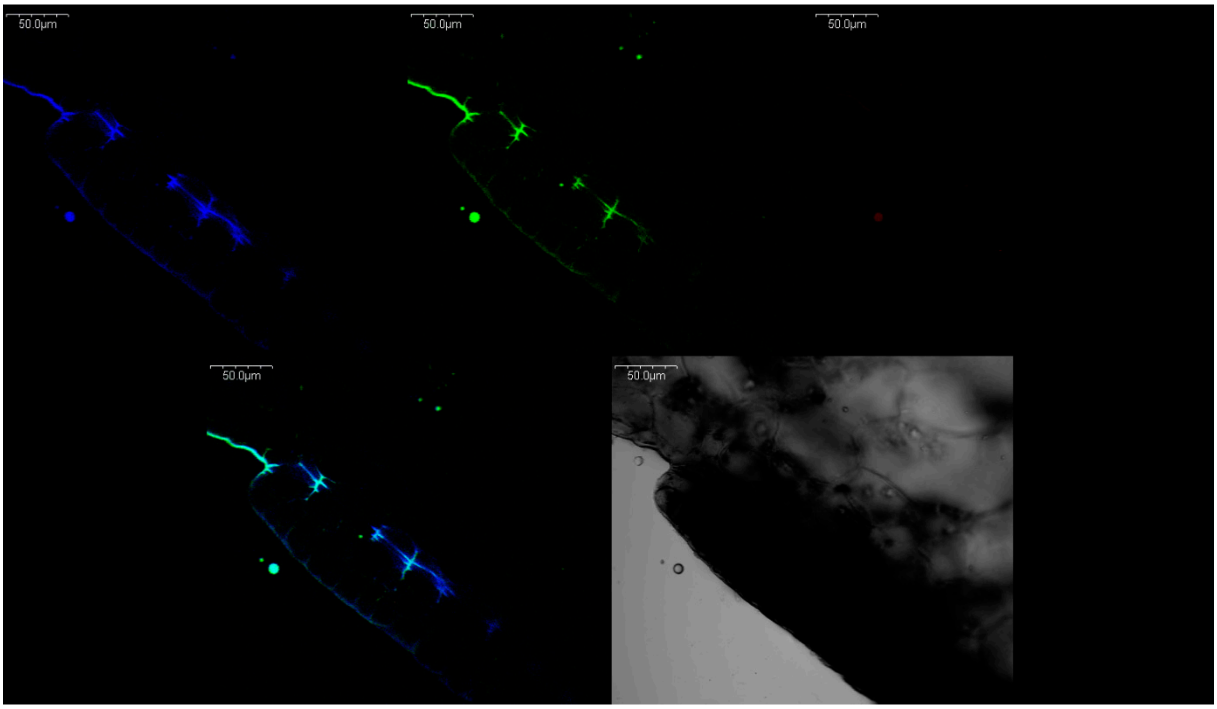

Figure S9
Autofluorescence of unstained cross-sections from the glandular zone of pitchers showing visible symptoms of senescence (aged).

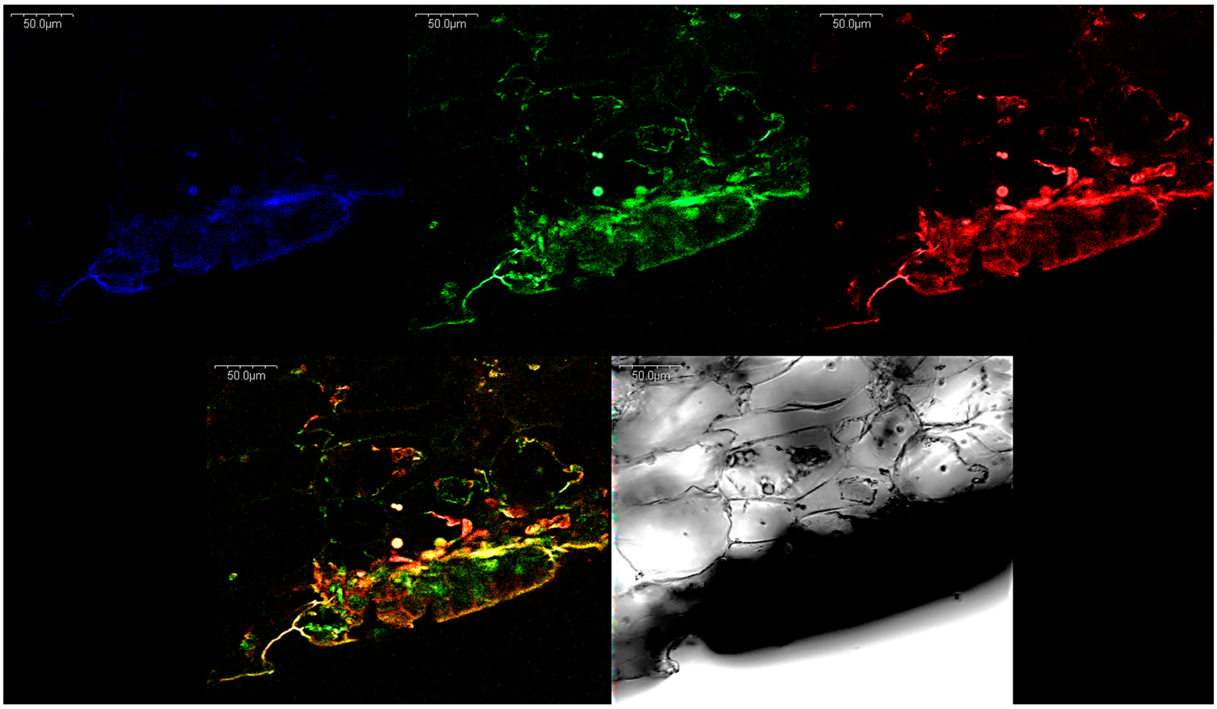

Figure S10
Fluorescence of DPBA-stained cross-sections from the glandular zone of aged pitchers showing visible symptoms of senescence.

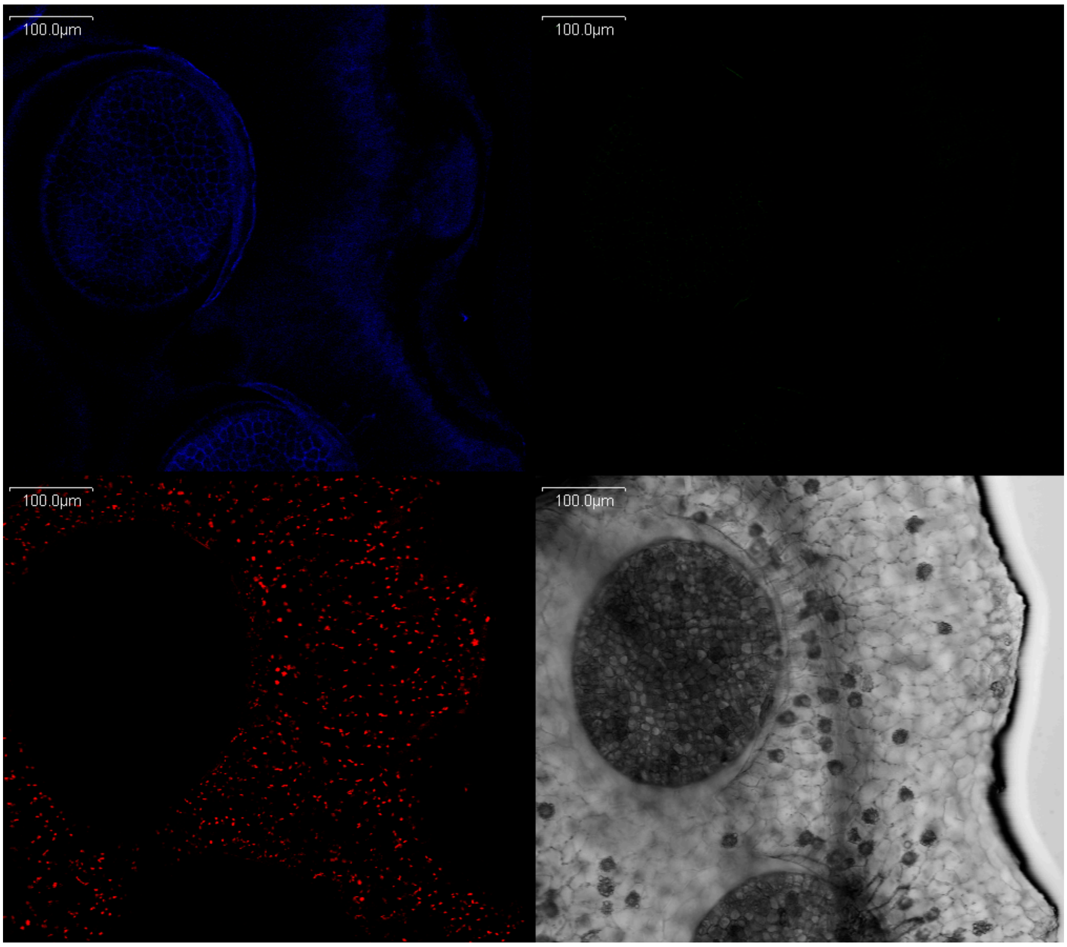
100.0μm
100.0μm
100.0μm
100.0μm

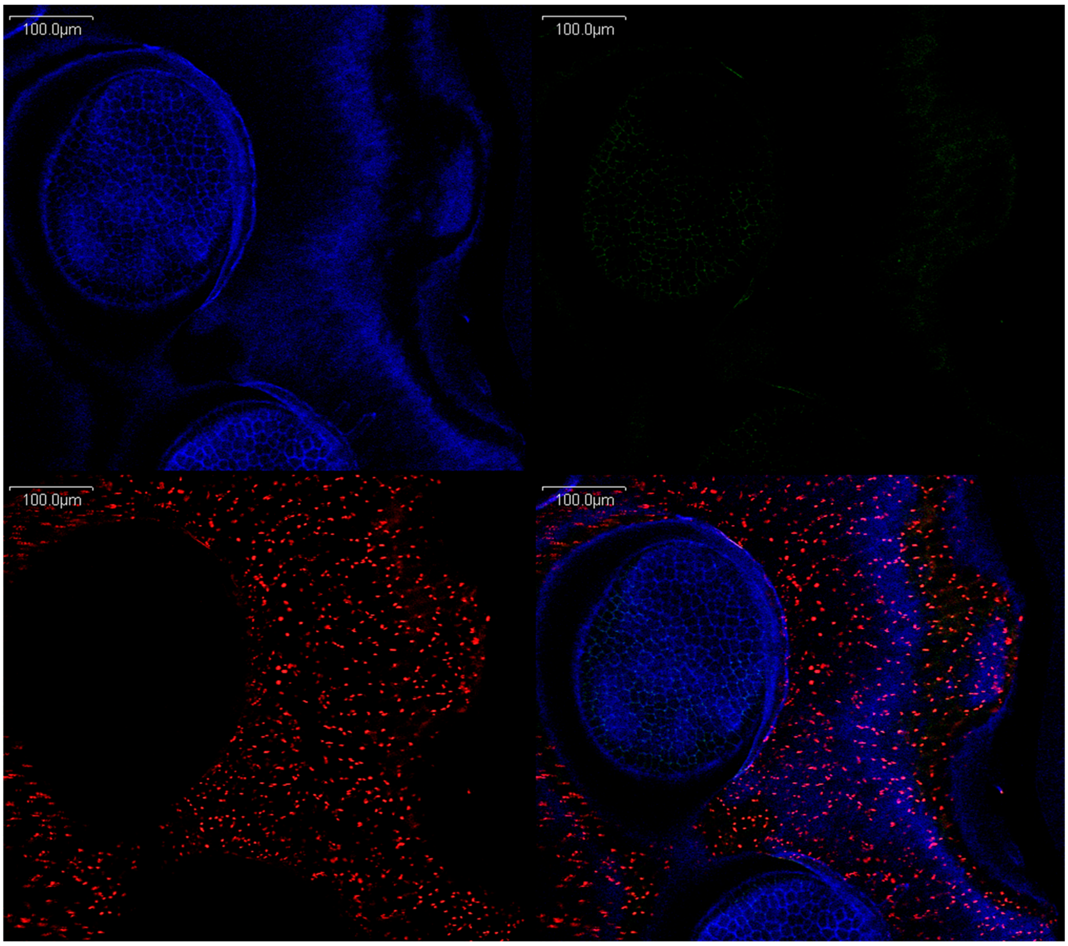

Figure S11
Autofluorescence of unstained epidermal peels from the glandular zone of closed pitchers.

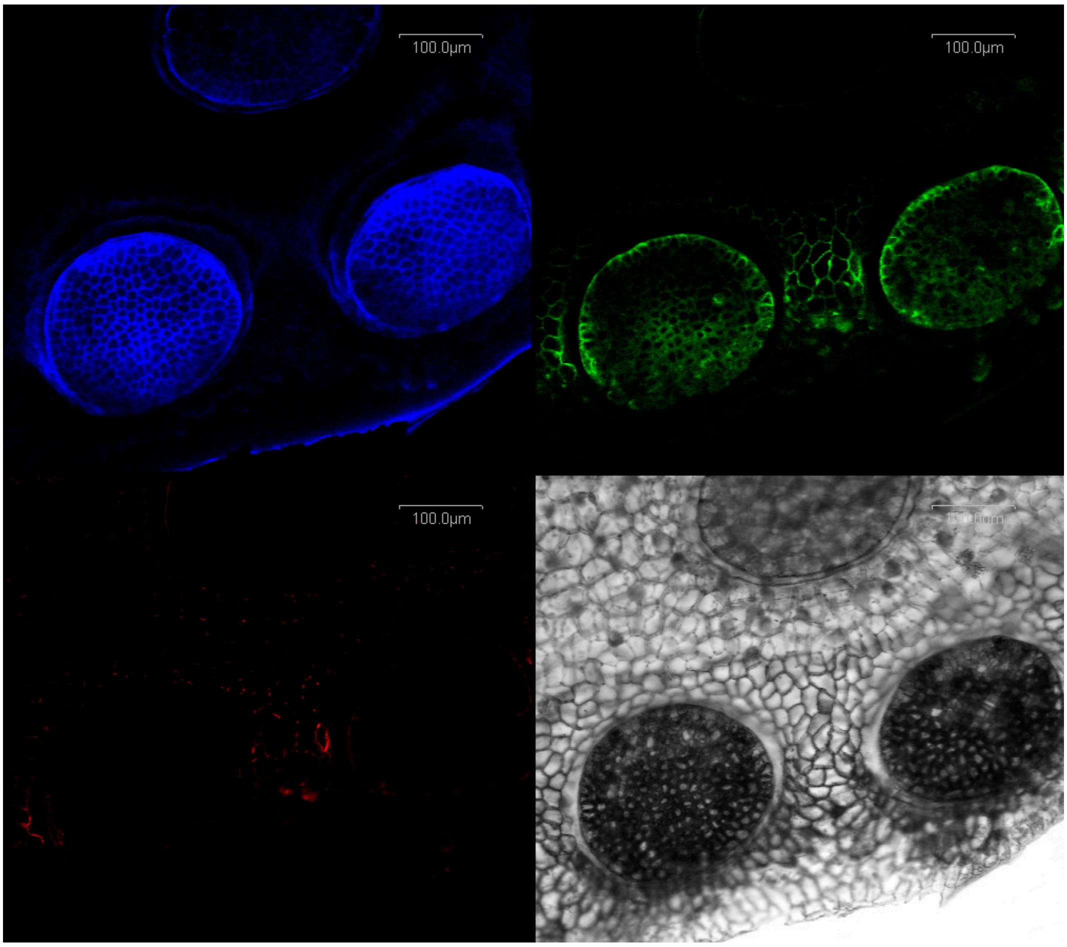
100.0µm
100.0µm
100.0µm

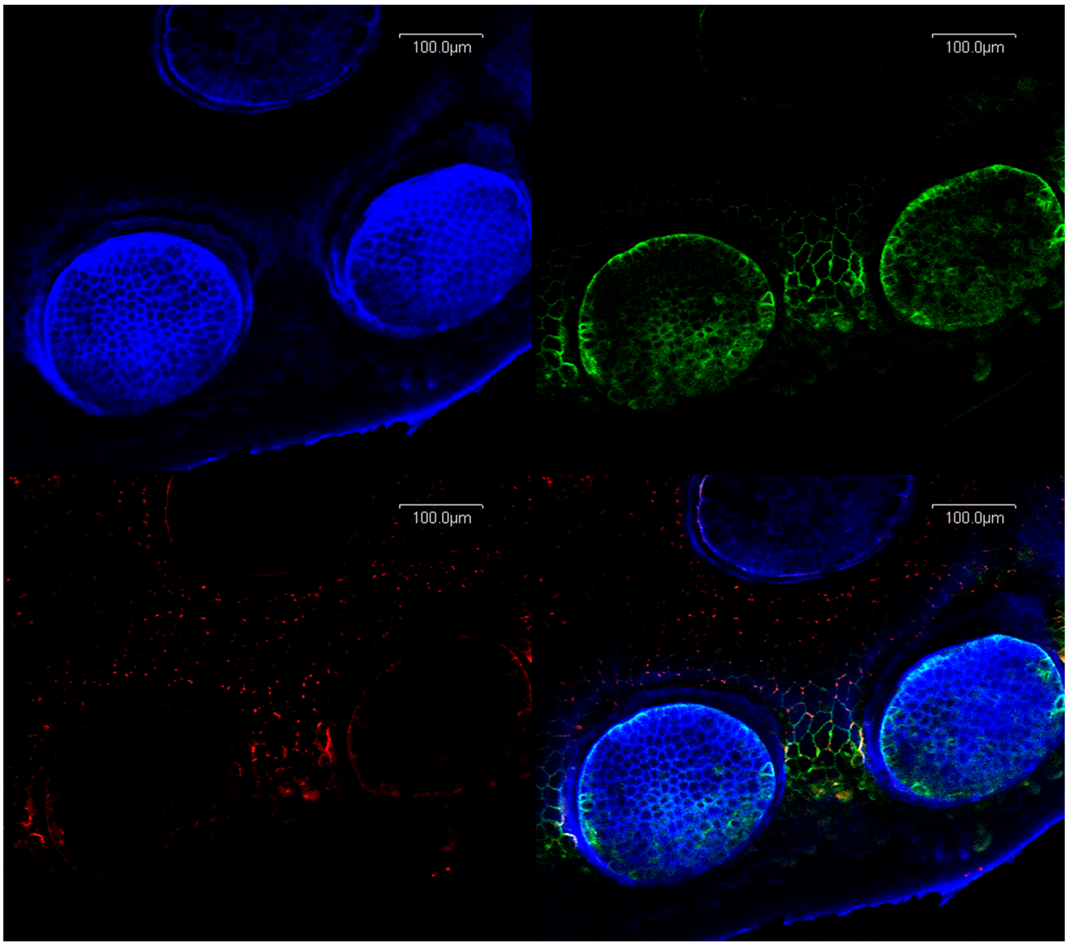

Figure S12

Fluorescence of DPBA-stained epidermal peels from the glandular zone of closed pitchers.

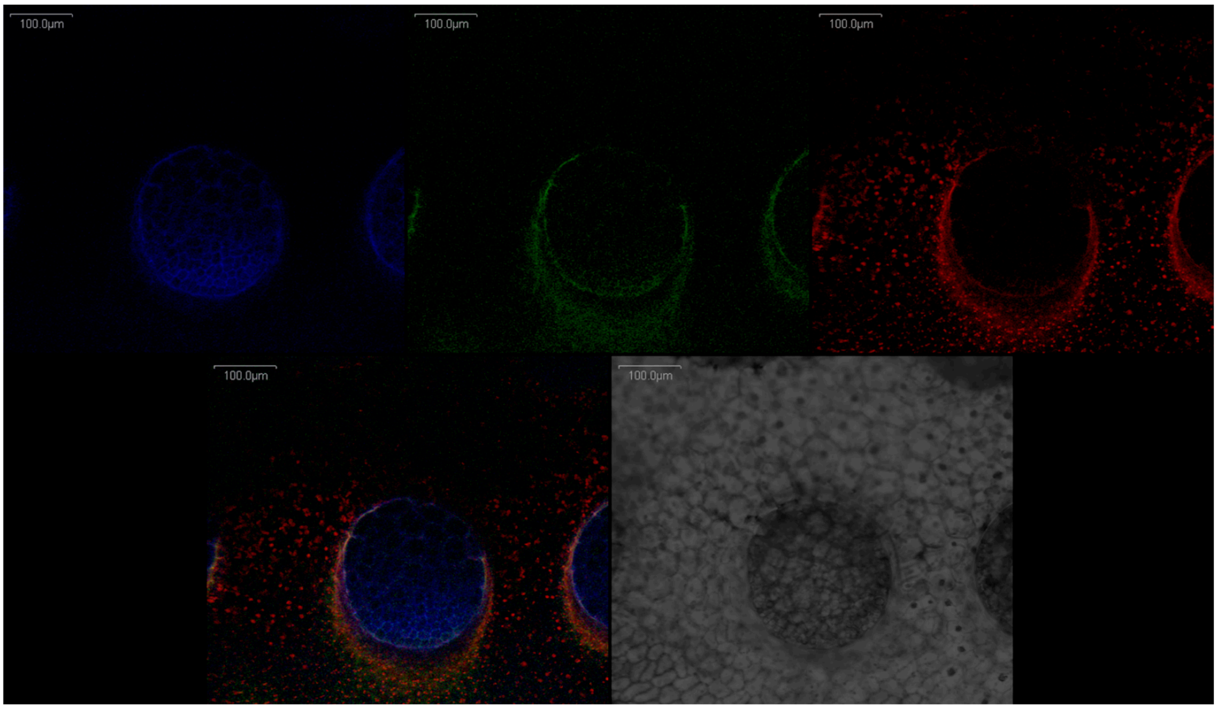

Figure S13

Autofluorescence of unstained epidermal peels from the glandular zone of freshly opened immature pitchers.

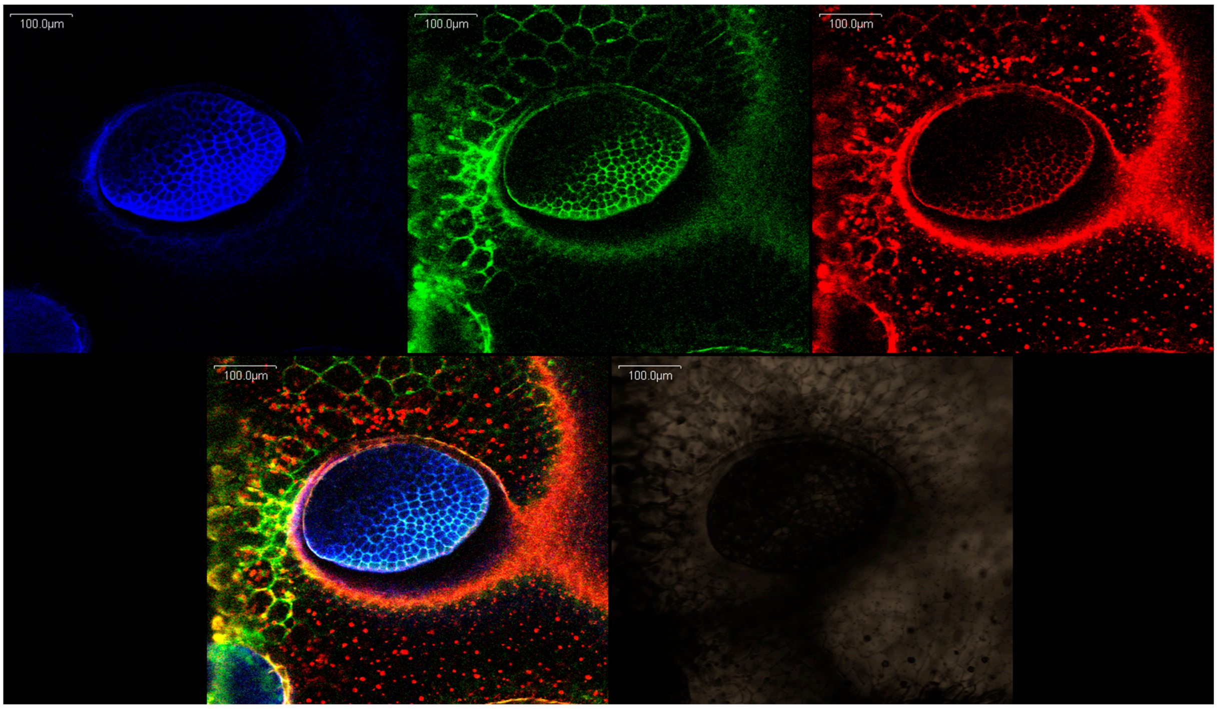

Figure S14

Fluorescence of DPBA-stained epidermal peels from the glandular zone of freshly opened immature pitchers.

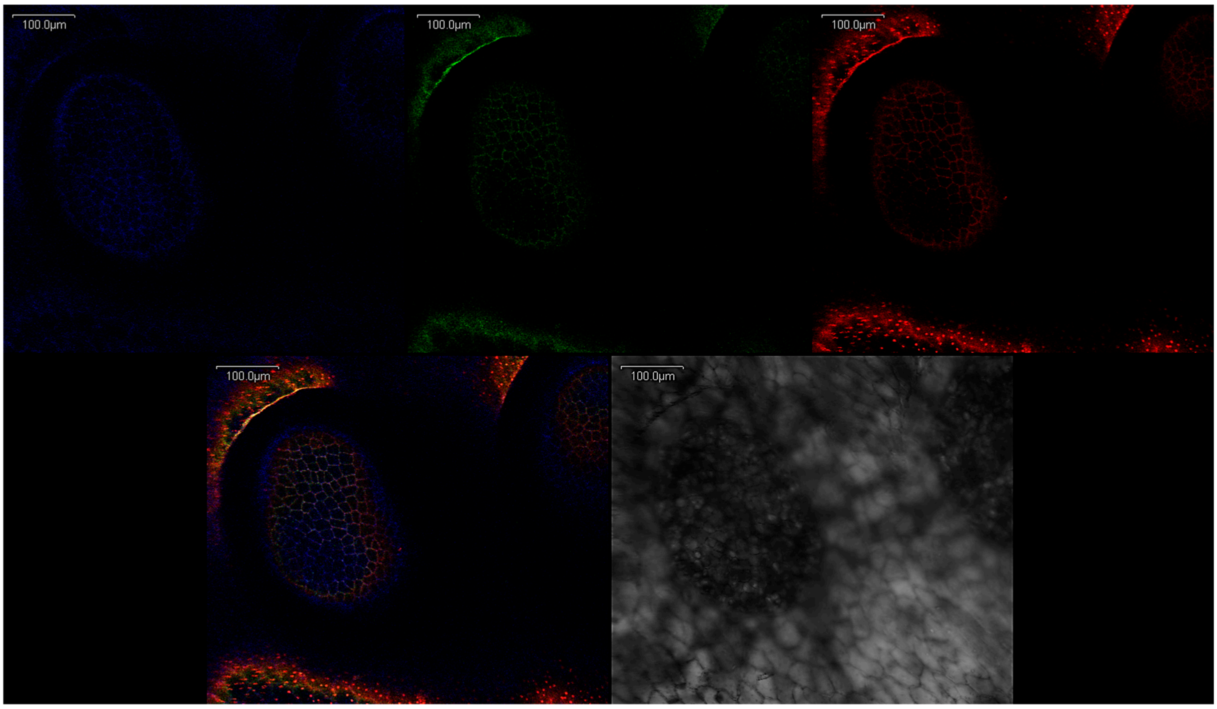

Figure S15

Autofluorescence of unstained epidermal peels from the glandular zone of mature non-fed pitchers.

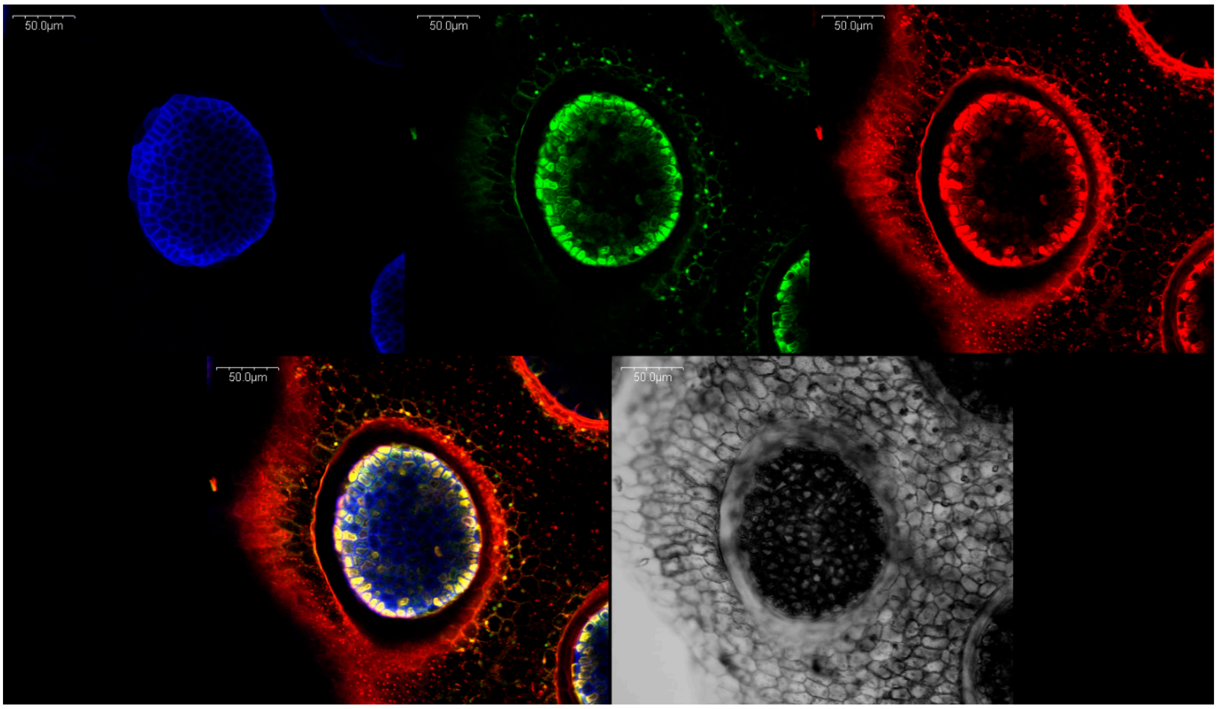

Figure S16

Fluorescence of DPBA-stained epidermal peels from the glandular zone of mature non-fed pitchers.

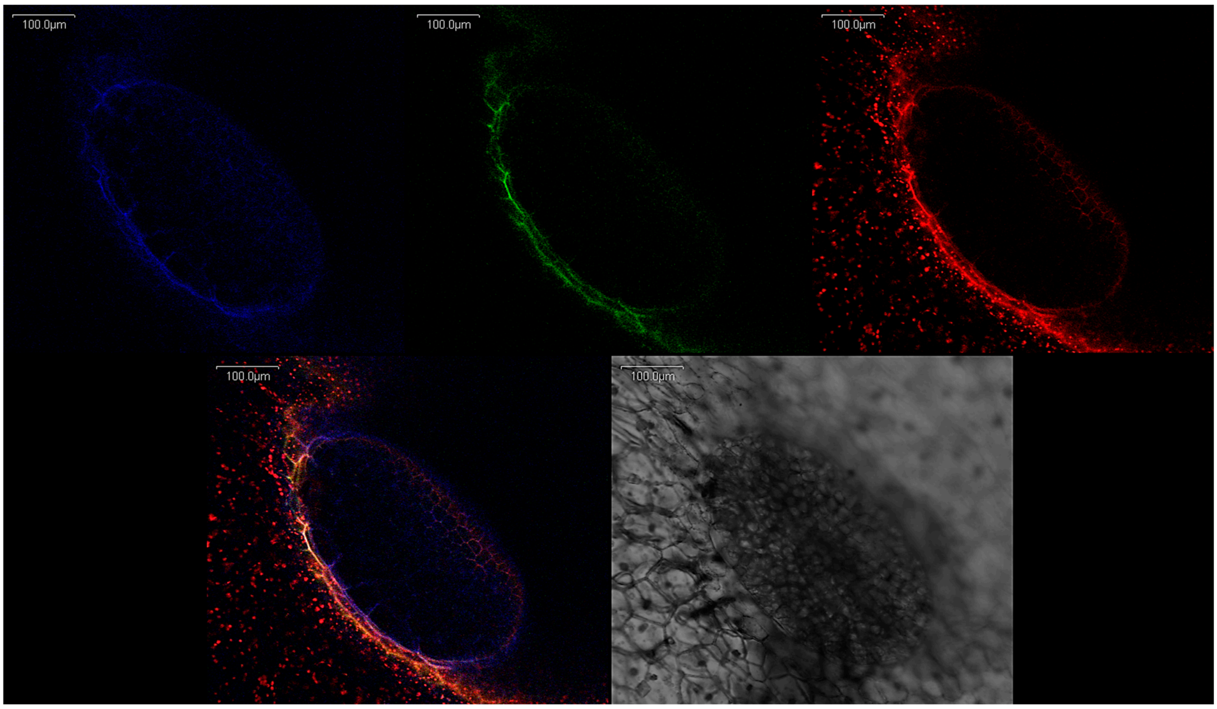

Figure S17

Autofluorescence of unstained epidermal peels from the glandular zone of mature fed pitchers.

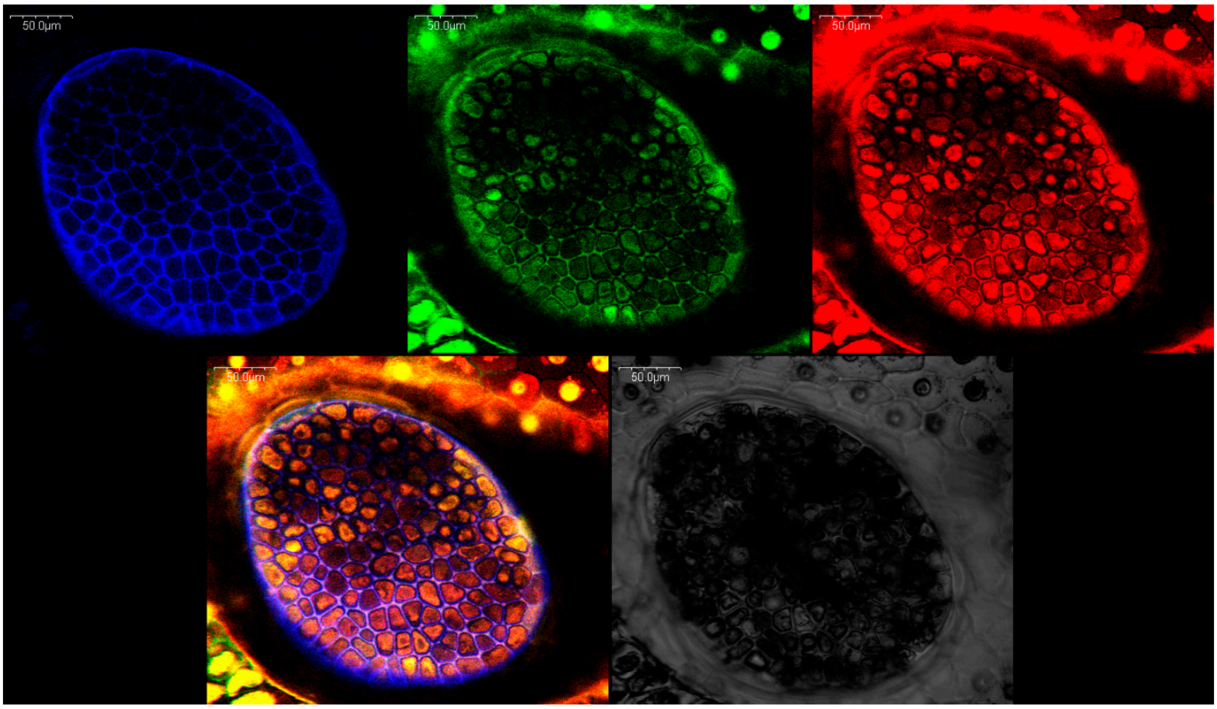

Figure S18

Fluorescence of DPBA-stained epidermal peels from the glandular zone of mature fed pitchers.

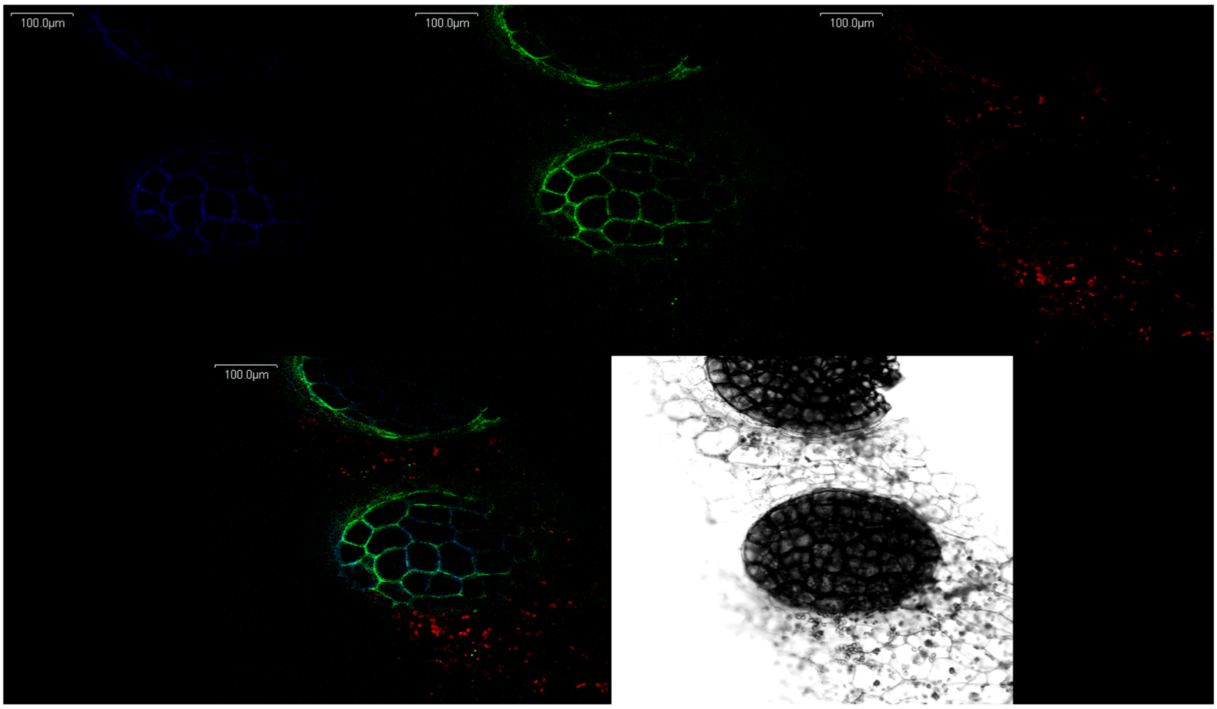

Figure S19

Autofluorescence of unstained epidermal peels from the glandular zone of aged pitchers showing visible symptoms of senescence (aged).

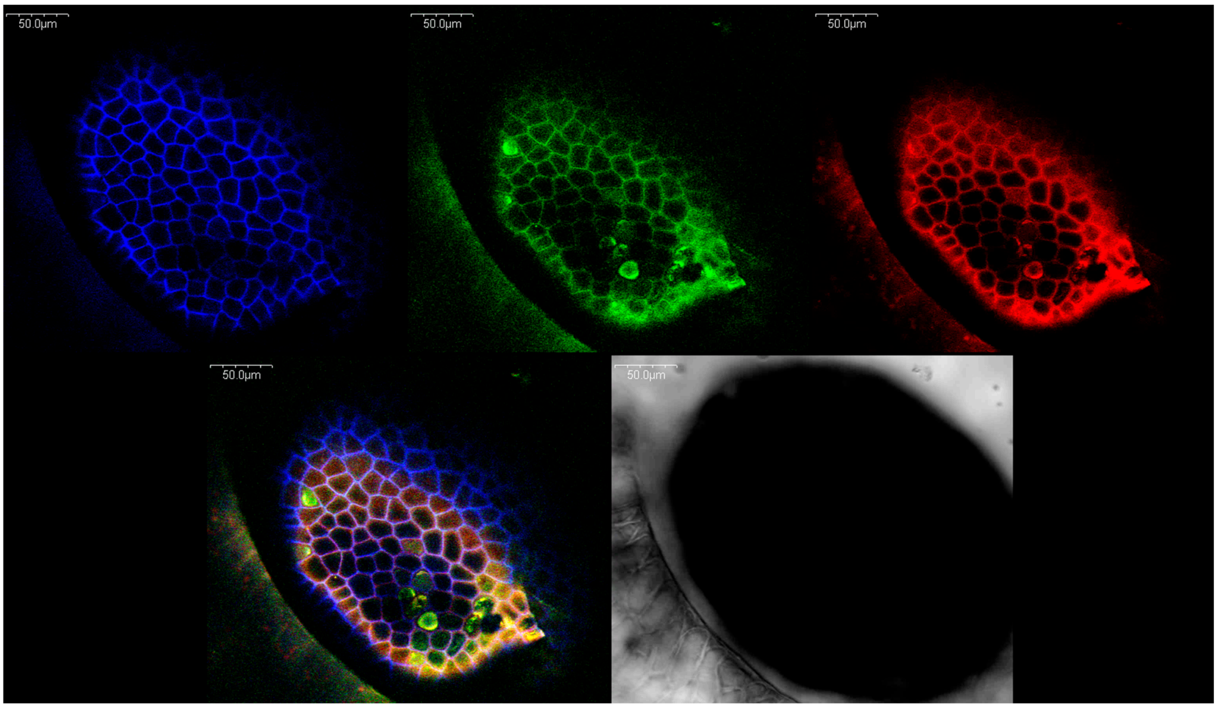

Figure S20

Fluorescence of DPBA-stained epidermal peels from the glandular zone of aged pitchers showing visible symptoms of senescence (aged).
